# Smart Mesoporous Silica Nanoparticle-Based Drug Delivery Systems: Recent Advances in Biomedical Applications, Wound Healing and Therapeutic Perspectives

**DOI:** 10.3390/pharmaceutics18081044

**Published:** 2026-08-21

**Authors:** Manickam Rajkumar, Nadarajan Prathap, Vivekanand Ankush Kashid, Bhupendra G. Prajapati, Kokila Palani, Parappurath Narayanan Sudha, Prabhakaran Rajkumar, Biswajit Basu

**Affiliations:** 1 Department of Biotechnology, Karpagam Academy of Higher Education (Deemed to be University), Coimbatore 641 021, Tamil Nadu, India; 2 Center for Cancer Research, Karpagam Academy of Higher Education (Deemed to be University), Coimbatore 641 021, Tamil Nadu, India; 3 Department of Science and Technical Development, Manash Kozybayev North Kazakhstan University, Petropavlovsk 150000, Kazakhstan; 4 MABD Institute of Pharmaceutical Education and Research, Babhulgaon, Yeola, Nashik 423 401, Maharashtra, India; 5 Center for Research Impact & Outcome, Chitkara College of Pharmacy, Chitkara University, Rajpura 140 401, Punjab, India; 6 Department of Pharmaceutics, Parul Institute of Pharmacy, Faculty of Pharmacy, Parul University, Waghodia, Vadodara 391 760, Gujarat, India; 7 Department of Industrial Pharmacy, Faculty of Pharmacy, Silpakorn University, Nakhon Pathom 73000, Thailand; 8 Department of Pharmaceutical Engineering, Center for Drug Discovery and Development, Vinayaka Mission’s Kirupananda Variyar Engineering College, Vinayaka Mission’s Research Foundation, Salem 636 308, Tamil Nadu, India; 9 Department of Physiology, Saveetha Dental College & Hospitals, Saveetha Institute of Medical and Technical Sciences (SIMATS), Saveetha University, Chennai 600 077, Tamil Nadu, India; 10 School of Applied Bioscience, Food and Agritech, Rathinam Global Deemed to be University, Coimbatore 641 021, Tamil Nadu, India; 11 Department of Pharmaceutical Technology, School of Health & Medical Sciences, Adamas University, Barasat, Kolkata 700 126, West Bengal, India

**Keywords:** mesoporous silica nanoparticles, drug delivery systems, stimuli-responsive nanocarriers, tissue engineering, wound healing

## Abstract

Mesoporous silica nanoparticles (MSNs) have emerged as versatile nanocarriers for biomedical applications because of their unique physicochemical properties, including high surface area, large pore volume, excellent drug-loading capacity, controllable biodegradation, and facile surface functionalization. These characteristics have enabled the development of advanced drug delivery systems with enhanced therapeutic efficacy, targeted delivery, improved bioavailability, and reduced systemic toxicity. Recent advances in MSN synthesis, physicochemical properties, surface engineering, and functionalization strategies have significantly improved their biological performance and therapeutic potential. In particular, integrating polymers, lipids, and liposomes with MSN platforms has enhanced colloidal stability, circulation time, cellular uptake, and target specificity, thereby facilitating efficient, stimuli-responsive drug delivery. This review highlights MSN-based drug delivery systems in cancer therapy, where multifunctional nanocarriers enable site-specific delivery, controlled drug release, enhanced tumor accumulation, and reduced off-target effects. The review discusses the expanding roles of MSNs in antimicrobial therapy, wound healing, tissue engineering, and regenerative medicine, emphasizing their ability to promote localized therapeutic delivery, immunomodulation, angiogenesis, and tissue regeneration. The review discusses the diagnostic and theragnostic capabilities of MSNs for disease imaging and monitoring. It also critically evaluates current challenges related to biocompatibility, biodegradation, toxicity, biological barriers, large-scale manufacturing, clinical translation, and regulatory considerations. This review provides a comprehensive overview of recent progress, current limitations, and future opportunities for MSN-based platforms in targeted drug delivery and advanced biomedical applications, supporting their continued advancement toward clinical translation and precision medicine.

## 1. Introduction

Recent progress in materials science and nanotechnology has significantly advanced the development and application of nanomaterials in fields such as medicine, agriculture, and engineering. Nanomaterials have attracted considerable attention for biomedical applications due to their unique physicochemical characteristics, including a high surface-area-to-volume ratio, tunable surface functionality, and nanoscale dimensions [1]. Compared with inorganic nanomaterials, mesoporous silica nanoparticles (MSNs) are particularly promising for clinical and translational research because of their remarkable biocompatibility, stability, and therapeutic potential. Nanoparticles address the disadvantages of conventional treatment methods, such as limited bioavailability, nonspecific distribution, and uncontrolled drug release, serving as effective drug-delivery platforms [2]. Additionally, by improving target specificity and therapeutic retention, nanomaterial-based delivery systems increase the effectiveness of antimicrobial medicines. Through regulated, stimulus-responsive medication release, nanomaterials in regenerative medicine enhance tissue repair and regeneration of bone, cartilage, and skin by serving as flexible carriers for bioactive compounds. Overall, these advancements highlight the important role that nanomaterials play in improving therapeutic accuracy, overcoming biological barriers, and enhancing outcomes in wound healing, tissue engineering, and cancer treatment [3,4].

Among nanomaterials, MSNs have attracted considerable interest due to their unique structural and physicochemical properties. MSNs are usually made from amorphous silica and have a highly organized mesoporous structure made up of silicate tetrahedra joined by stable covalent bonds. MSNs are especially well-suited for biomedical applications such as drug delivery, tumor targeting, bioimaging, and biosensing because of their large specific surface area, high pore volume, and drug-loading capacity [5]. Although organic nanocarriers typically exhibit passive drug release and low targeting efficiency, this can sometimes limit their therapeutic utility. In contrast, silane-based surface modification of MSNs enables gate-controlled, stimuli-responsive delivery systems with enhanced targeting capabilities [6]. Incorporating an internal porous reservoir with adjustable surface functions enables controlled drug release, active targeting, and multimodal therapeutic and diagnostic applications. Consequently, MSNs have become viable nanoplatforms for sophisticated biomedical therapies and precision medicine [7].

Mesoporous silica nanoparticles are among the most extensively studied inorganic nanocarriers for advanced drug delivery due to their highly ordered mesoporous structure, large specific surface area, pore size, high pore volume, robust physicochemical stability, and favorable biocompatibility [8]. These structural characteristics enable continuous or controlled medication release, high loading capacity, effective drug encapsulation, and protection of therapeutic substances from premature degradation [9]. The abundance of surface silanol groups enables functionalization with polymers, biomolecules, targeted ligands, and responsive gatekeepers. This allows for precise regulation of cellular uptake, biodistribution, and pharmacokinetic profiles. Advancements in nanotechnology have enabled precise control over MSN size, shape, pore architecture, and surface chemistry, aiding the development of drug delivery systems tailored to specific diseases [10]. As a result, MSNs have demonstrated great promise for delivering small-molecule medications, proteins, peptides, nucleic acids, growth factors, and imaging agents across a range of biomedical sectors, including cancer therapy, wound healing, antimicrobial treatment, tissue engineering, and regenerative medicine [11].

Conventional MSNs offer several benefits, but their limitations include nonspecific biodistribution, premature drug leakage, poor control of drug release, and reduced therapeutic efficacy in complex physiological settings [12]. To overcome these challenges, researchers have incorporated stimuli-responsive materials, targeting ligands, polymers, peptides, aptamers, and imaging probes into the MSN structure to create Smart Mesoporous Silica Nanoparticles (Smart MSNs). These sophisticated nanoplatforms enable site-specific and controlled drug release by selectively responding to exogenous triggers such as light, magnetic fields, ultrasound, and temperature, as well as endogenous stimuli such as pH, reactive oxygen species (ROS), redox potential, enzymes, hypoxia, and ATP [13]. Smart MSNs are multifunctional theragnostic platforms that enable targeted therapy, bioimaging, biosensing, gene delivery, and combination treatments in addition to regulated drug administration. Smart MSNs are promising for precision medicine and future clinical applications because they can enhance therapeutic efficacy, increase tissue-specific accumulation, extend systemic circulation, bypass biological barriers, and reduce systemic toxicity [14].

Smart nanocarrier-based drug delivery systems have advanced significantly, with lipid nanoparticles (LNPs), liposomes, polymeric nanoparticles (PNPs), dendrimers, inorganic nanoparticles, and MSNs as the primary classes of delivery platforms. Every system has unique benefits and drawbacks that affect its suitability for different biomedical applications [15]. Liposomes and LNPs have successfully delivered nucleic acids and small compounds in clinical settings, and both are biocompatible. Nevertheless, these platforms frequently show poor control over prolonged drug release, decreased drug-loading capacity for certain treatments, and limited structural stability [16]. For instance, PNPs offer superior biodegradability and flexible surface modification, yet they may exhibit batch-to-batch variability, complex synthesis procedures, and limited capacity to load hydrophilic agents. MSNs, by contrast, have a highly organized mesoporous structure, large pore volume, variable pore sizes, high surface area, strong physicochemical stability, and numerous surface silanol groups [17]. These features allow for varied surface functionalization, regulated or stimuli-responsive release, and effective drug encapsulation. Despite these benefits, MSNs still face many obstacles compared with clinically proven lipid-based systems. These obstacles include protein corona formation, uptake by the mononuclear phagocyte system, long-term biodegradation, manufacturing uniformity, and regulatory approval. As a result, MSNs offer a distinctive platform by combining multifunctional engineering with high loading capacity, making them attractive options for precision nanomedicine, theragnostics, and targeted drug delivery [18]. To improve their clinical translation, additional research is required.

MSNs have shown great promise as flexible nanocarriers for targeted drug delivery and advanced biomedical applications. MSNs are frequently surface-functionalized with biological macromolecules such as monoclonal antibodies, peptides, aptamers, polymers, nucleic acids, and other targeting ligands to enhance targeting specificity, therapeutic efficacy, and controlled drug release [19]. These changes improve tissue-specific targeting, receptor-mediated cellular uptake, and therapeutic efficacy. However, they also increase nanoparticle hydrodynamic diameter, molecular weight, and surface complexity, which significantly affect pharmacokinetic characteristics. Variations in particle size, surface charge, and ligand density can affect blood circulation duration, biodistribution, vascular extravasation, tissue penetration, cellular internalization, biodegradation, and clearance pathways [20]. Additionally, larger, more thoroughly functionalized MSNs are more likely to form a protein corona, undergo opsonization, and be recognized by the mononuclear phagocyte system (MPS), resulting in preferential accumulation in the liver, spleen, and other reticuloendothelial organs. In addition to increasing the likelihood of complement activation, macrophage-mediated clearance, inflammatory cytokine release, hypersensitivity reactions, and longer tissue retention, this phenomenon can reduce tumor or tissue-specific delivery [21]. Therefore, to balance targeting efficiency, pharmacokinetics, biosafety, and successful clinical translation of MSN-based nanotherapeutics, careful optimization of particle size, surface chemistry, ligand density, and biodegradable functional coatings is essential.

MSN-based drug delivery has advanced significantly, but most of the reviews have focused on limited facets of MSN research, such as synthesis, drug-loading techniques, cancer treatment, or general biomedical applications. In contrast, the integration of intelligent, multipurpose MSN platforms and the challenges of clinical translation have received relatively little attention. Furthermore, few systematic comparative studies have evaluated surface functionalization techniques, stimuli-responsive gatekeepers, targeted drug delivery mechanisms, pharmacokinetics, biodegradation, long-term biosafety, manufacturing scalability, and regulatory considerations. As a result, a significant gap remains between encouraging preclinical findings and the effective clinical application of MSN-based nanomedicines. This review addresses this gap by providing a current, integrated perspective on nanocarrier-based Smart MSN drug delivery systems, with a comprehensive examination of their physicochemical design, surface engineering, stimuli-responsive release mechanisms, and targeted delivery strategies. This review also critically evaluates recent developments in biomedical and theragnostic applications, including cancer treatment, antibiotic therapy, wound healing, tissue engineering, and regenerative medicine. This review discusses key biological and translational issues, including immunological interactions, pharmacokinetics, biodistribution, biodegradation, manufacturing, and regulatory barriers. This review offers a thorough framework and future views to guide the logical development, optimization, and clinical use of next-generation Smart MSN-based nanomedicines.

## 2. Synthesis and Properties of MSNs

### 2.1. Synthesis of MSNs

Researchers can precisely control the structural and physicochemical features of MSNs using a variety of synthesis techniques. Because of its ease of use, adaptability, and repeatability, the surfactant-assisted sol–gel approach is the most popular. This process creates highly structured mesoporous frameworks by hydrolyzing and condensing silica precursors like tetraethyl orthosilicate (TEOS) or tetramethyl orthosilicate (TMOS) in the presence of structure-directing surfactants [22]. Modifying synthesis parameters such as pH, temperature, precursor concentration, solvent composition, and surfactant type can adjust MSNs’ physicochemical features, including particle size, pore diameter, pore volume, morphology, and surface characteristics. After removing the surfactant template, a distinct porous structure with a large surface area and a consistent mesopore distribution forms [23].

To fabricate MSNs with diverse structural characteristics, studies have developed sophisticated manufacturing techniques beyond traditional sol–gel synthesis. Evaporation-induced self-assembly arranges silica-surfactant assemblies into ordered mesostructures using regulated solvent evaporation [24]. Hard-templating techniques use stiff sacrificial templates to achieve exact control over particle geometry and pore organization. In contrast, soft-templating techniques use self-assembled amphiphilic aggregates to fabricate mesoporous architectures under mild circumstances [25]. These techniques can be used to analyze MSNs with a variety of morphologies, such as spherical, rod-shaped, hollow, core–shell, and hierarchical architectures. Multiple synthesis techniques allow customization of MSN structural features, surface chemistry, porosity, and overall physicochemical properties to meet specific design requirements [26].

### 2.2. Physicochemical Properties of MSNs

MSNs differ from conventional nanocarrier systems due to a unique mix of structural and physicochemical properties. Their highly ordered mesoporous architecture, high specific surface area, increased pore volume, adjustable pore diameter, and strong physicochemical stability allow effective encapsulation and protection of a variety of medicinal compounds [27]. The interconnected pore network facilitates controlled release, reduces early drug leakage, and enables high loading capacity. Furthermore, the siloxane framework strongly resists thermal and chemical degradation, preserving structural integrity in physiological settings [28]. Careful control of MSN nanoscale particle size can affect cellular interactions, biodistribution, circulation dynamics, and overall biological performance. Additionally, customizable pore diameters allow incorporation of a variety of payloads, such as proteins, peptides, nucleic acids, small-molecule drugs, and other bioactive molecules [29].

The remarkable tunability of MSNs in size, shape, porosity, surface chemistry, and degradation profiles are another important benefit. Surface silanol groups are abundant, making it easy to functionalize them with polymers, biomolecules, targeting ligands, and stimuli-responsive components [30]. This facilitates the development of multifunctional nanoplatforms with enhanced biological efficacy. Furthermore, MSN morphology can be modified into a variety of structures, including hollow, core–shell, spherical, rod-shaped, and hierarchical forms, each with distinct interactions with biological systems [31]. This ability to modify physicochemical attributes enables precise control over cargo loading, release kinetics, cellular uptake, and biodistribution. MSNs are among the most promising and versatile platforms in nanomedicine and drug-delivery research because they integrate a large surface area, adjustable porosity, structural stability, programmable shape, and diverse surface modifications [32].

### 2.3. Functionalization Strategies of MSNs

The release mechanism, biological stability, and clinical translatability of MSN-based drug delivery systems are greatly influenced by the selected surface functionalization technique, which also improves drug loading and targeting capabilities. Depending on the particular triggering stimulus, stimuli-responsive gatekeepers exhibit different release mechanisms and therapeutic characteristics [33]. To release drugs quickly in pathologically acidic environments, pH-responsive devices use acid-labile bonds or pH-sensitive polymers that cleave or swell in acidic tumor tissues, infected wounds, or intracellular endosomal and lysosomal compartments. To improve intracellular specificity and reduce premature extracellular drug release, redox-responsive methods typically employ disulfide linkages that are specifically cleaved by elevated intracellular glutathione concentrations in cancer cells [34]. To achieve extremely selective medication release, enzyme-responsive gatekeepers rely on disease-associated enzymes such as proteases, hyaluronidase, or matrix metalloproteinases. However, enzyme expression levels, which may differ significantly between patients and disease stages, affect therapeutic efficacy. Surface modification techniques also have distinct translational advantages and drawbacks. Although covalent conjugation often requires multistep synthesis, thorough characterization, and increased manufacturing complexity, it delivers strong chemical stability, prolonged circulation time, less premature drug leakage, and improved in vivo durability [35]. Physical adsorption, by contrast, offers a simple, economical, and scalable manufacturing process with high drug-loading efficiency; however, poor noncovalent interactions may lead to early drug release, reduced biological stability, and decreased reproducibility under physiological conditions [36]. Therefore, to enable the successful clinical translation of MSN-based nanomedicines, the optimal functionalization strategy must balance therapeutic efficacy, biological stability, manufacturing feasibility, and regulatory constraints.

### 2.4. Challenges of MSNs in Biomedical Applications

Despite the substantial therapeutic promise of MSNs, several biological obstacles hamper their clinical translation. Unmodified MSNs exhibit colloidal instability and rapidly adsorb plasma proteins upon systemic delivery, forming a protein corona that alters their biological identity and promotes opsonization [37]. Consequently, the MPS effectively identifies these nanoparticles, hastening their removal from circulation and causing preferential accumulation in reticuloendothelial organs such as the liver, spleen, and lungs. In addition, this nonspecific biodistribution increases the risk of off-target toxicity and adverse immunological reactions and reduces the medication’s availability at the intended site of action [38]. Advanced surface engineering methods, including PEGylation, zwitterionic polymers, biomimetic cell membrane coatings, protein corona modulation, surface charge optimization, and ligand-mediated active targeting, have been studied to overcome these issues [39]. Together, these tactics reduce MPS recognition, prolong circulation, improve colloidal stability, promote tissue-specific accumulation, and significantly enhance the therapeutic efficacy and translational potential of MSN-based drug delivery systems.

Long-term biodegradation and biosafety of MSNs also pose serious obstacles to therapeutic use, in addition to immune detection and systemic clearance. The hydrolysis of siloxane (Si–O–Si) bonds in silica nanoparticles yields biocompatible orthosilicic acid; however, the degradation rate depends on crystallinity, surface functionalization, silica condensation, pore architecture, and particle size [40]. After repeated delivery, highly condensed or extensively functionalized MSNs may accumulate and persist in tissue for extended periods due to their slow degradation. Persistent nanoparticles in organs such as the liver and spleen may jeopardize long-term biosafety by causing fibrosis, complement activation, oxidative stress, chronic inflammation, macrophage activation, and organ-specific toxicity [41]. A study has focused on creating biodegradable hybrid polymer-silica materials, improving particle size and porosity, adding environmentally friendly linkers, and developing biodegradable polymer coatings to accelerate nanoparticle breakdown and removal, thereby reducing these risks [42]. To safely translate MSN-based nanotherapeutics into clinical settings, extensive long-term investigations of pharmacokinetics, biodegradation, biodistribution, and repeated-dose toxicity are necessary.

## 3. MSN-Based Drug Delivery Systems

Drug delivery systems are essential for improving pharmaceutical efficacy by enhancing drug stability, bioavailability, controlled release, and site-specific targeting while reducing adverse effects. Because of their unique physicochemical and structural characteristics, MSNs have become a promising drug-delivery platform among the many nanocarriers developed [43]. MSNs’ large specific surface area, high pore volume, tunable pore size, and flexible surface chemistry enable effective loading and preservation of a variety of therapeutic agents, such as small-molecule medications, proteins, peptides, nucleic acids, and growth factors. MSNs can also overcome the drawbacks of traditional drug delivery methods, including poor solubility, rapid degradation, nonspecific distribution, and low therapeutic efficacy, thanks to their ability to precisely control particle size, morphology, porosity, and surface functionality [44]. Surface modifications with polymers, targeting ligands, and stimuli-responsive elements further enable controlled, targeted drug release in response to specific biological signals. As a result, MSN-based drug delivery systems have garnered significant interest as cutting-edge nanoplatforms to enhance therapeutic outcomes and advance precision medicine [45].

### 3.1. Drug Loading and Encapsulation Approaches

Drug loading and encapsulation efficiency are the main factors influencing the therapeutic efficacy of MSN-based drug delivery systems. Owing to their highly organized mesoporous structure, large specific surface area, and high pore volume, MSNs can accommodate substantial quantities of medicinal drugs with diverse physicochemical characteristics [46]. Depending on the properties of the therapeutic payload and the MSN surface, drug loading typically occurs via physical adsorption, electrostatic interactions, hydrogen bonding, covalent conjugation, or pore encapsulation. Pore size, pore volume, surface chemistry, particle shape, drug solubility, and interactions between drug molecules and the silica matrix are some of the variables that affect drug-loading efficiency [47]. MSNs are frequently functionalized with polymers, biomolecules, or stimuli-responsive gatekeepers that control cargo retention and release, thereby increasing loading capacity and preventing early drug leakage. By enabling regulated, site-specific release patterns and increasing encapsulation efficiency and drug stability, these techniques maximize therapeutic efficacy while reducing systemic toxicity [48]. Therefore, developing sophisticated MSN-based drug delivery systems depends on the logical design of drug-loading and encapsulation techniques.

### 3.2. Controlled and Stimuli-Responsive Release

Controlled, sustained drug release is critical for improving therapeutic efficacy, reducing dosing frequency, and minimizing systemic adverse effects. Due to their large pore volume, high surface area, tunable pore architecture, and adaptable surface chemistry, MSNs have become highly effective drug-delivery platforms for controlled, site-specific therapeutic release [49]. The well-ordered mesoporous framework of MSNs supports high drug-loading capacity and enables sustained, predictable release kinetics, maintaining drug concentrations within the therapeutic window. Additionally, surface modification with polymers, biomolecules, targeting ligands, or molecular gatekeepers enables precise control of drug diffusion and release profiles [50]. Advanced MSN systems respond to endogenous stimuli such as pH, ROS, redox potential, and enzyme activity, as well as exogenous triggers including light, temperature, magnetic fields, and ultrasound. These stimuli-responsive systems facilitate on-demand drug release, increase drug accumulation at target sites, improve therapeutic precision, and reduce systemic toxicity, positioning MSNs as promising candidates for precision nanomedicine [51].

Although ROS- and pH-responsive MSN systems offer significant advantages, their therapeutic performance in in vivo environments is substantially more complex than under controlled in vitro conditions. Activation of these nanoplatforms requires pathological stimuli to reach threshold levels sufficient to induce gatekeeper dissociation or degradation and initiate controlled drug release [52]. The physiological microenvironment, however, is highly heterogeneous and dynamic, with considerable spatial and temporal variations in pH, ROS, redox potential, enzyme activity, oxygen availability, and inflammatory status across tissues, disease stages, and even within individual lesions. As a result, endogenous stimuli may be insufficient, heterogeneous, or transient, leading to unpredictable release kinetics, premature drug leakage, incomplete payload release, or delayed therapeutic activation [53]. In addition, biological barriers such as protein corona formation, opsonization, MPS-mediated clearance, extracellular matrix barriers, heterogeneous vascular permeability, elevated interstitial fluid pressure, and limited tissue penetration can further restrict nanoparticle accumulation and access to stimuli at target sites. These factors collectively contribute to discrepancies between in vitro and in vivo therapeutic outcomes [54]. Future research should prioritize developing highly sensitive, robust multi-stimuli-responsive MSN platforms with optimized activation thresholds, validated in clinically relevant animal models, supported by comprehensive pharmacokinetic and biodistribution studies, and evaluated for long-term biosafety to improve the reliability and clinical translatability of MSN-based nanomedicines.

### 3.3. Targeted and Multifunctional Drug Delivery Platforms

Targeted drug delivery enables enhanced therapeutic efficacy while reducing systemic toxicity and off-target effects. Because of their versatile surface functionalization, high loading capacity, and adjustable physicochemical characteristics, MSNs show great promise as targeted drug delivery systems. The abundance of surface silanol groups enables conjugation to a variety of targeted ligands, including antibodies, peptides, aptamers, polysaccharides, and small molecules [55]. This facilitates recognition and uptake by particular cells or tissues. In addition to active targeting, MSNs’ modifiable size and surface properties can be tailored to enhance biodistribution and prolong circulation. Additionally, MSNs can be designed as multifunctional nanoplatforms that integrate imaging probes, therapeutic agents, targeting moieties, and stimuli-responsive components into a single system [56]. This multifunctionality enhances treatment precision and improves therapeutic outcomes by enabling concurrent drug delivery, disease monitoring, and controlled therapeutic intervention. Targeted, multifunctional MSN-based systems are therefore a significant step toward next-generation nanomedicine platforms for individualized, precise healthcare. The ligand-free PEGylated MSN (RMSN25-PEG-TA), with a particle size of about 25 nm, exhibited prolonged systemic circulation and improved BBB penetration. With increased permeability and retention, DOX@RMSN25-PEG-TA achieved brain accumulation nearly sixfold higher than that of free doxorubicin. In vivo tests demonstrated significant suppression of orthotopic glioma growth, increased survival, and enhanced biosafety. Proteins including albumin and apolipoprotein E, which may aid BBB transit, were shown to be enriched in the nanoparticle protein corona, according to proteomic research [57].

**Figure 1:**
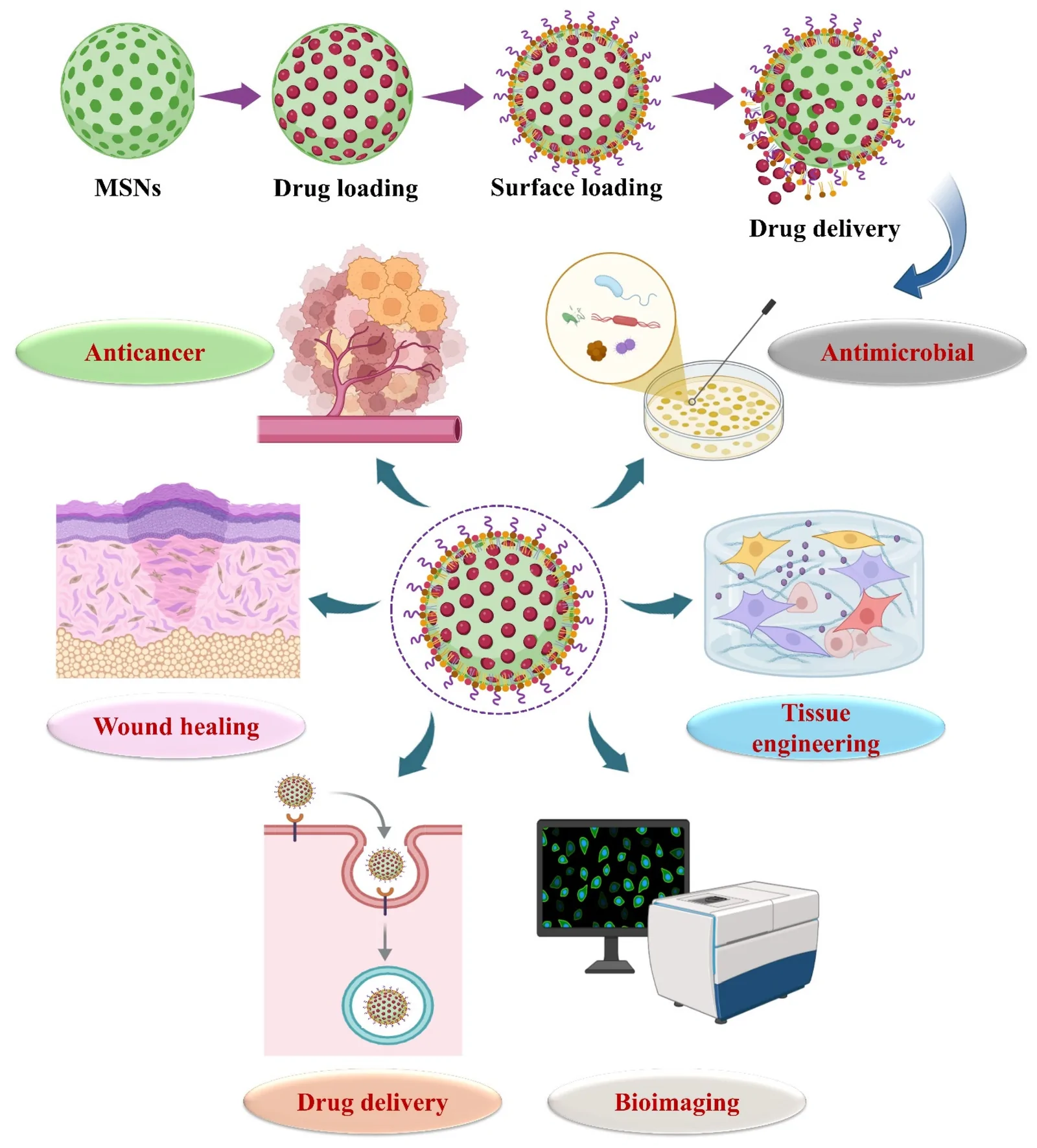
Schematic illustration of MSNs showing sequential drug loading, surface functionalization, and controlled drug delivery. The functionalized MSNs are highlighted for diverse biomedical applications including anticancer therapy, antimicrobial activity, wound healing, tissue engineering, bioimaging, and targeted drug delivery (created using BioRender.com).

MSNs have emerged as versatile nanoplatforms for a wide range of biomedical applications due to their unique physicochemical properties. The porous structure of MSNs can effectively encapsulate therapeutic substances, as seen in Figure 1. Surface modification with polymers, biomolecules, or targeted ligands further improves drug loading, stability, and controlled release [58]. By increasing therapeutic efficacy and reducing off-target toxicity, these modified nanocarriers show significant promise for targeted drug delivery. Functionalized MSNs have been extensively researched for anticancer therapy, antimicrobial treatment, wound healing, tissue engineering, and bioimaging in addition to drug delivery. Their ability to co-deliver multiple therapeutic cargoes and to respond to internal or external stimuli enables precise manipulation of biological processes. As a result, MSNs represent a viable multifunctional platform for next-generation nanomedicine and improved theragnostic applications [59].

Protein corona formation significantly influences MSNs’ in vivo efficacy and clinical translation. Plasma proteins rapidly adsorb onto the MSN surface upon exposure to biological fluids, forming a dynamic protein corona that imparts a new biological identity to the nanoparticles. Therefore, the composition and evolution of the adsorbed protein layer, along with the intrinsic physicochemical features of MSNs, determine their biological behavior [60]. The protein corona significantly affects opsonization, complement activation, macrophage recognition, cellular uptake, biodistribution, circulation time, BBB penetration, and targeting efficiency. As a result, receptor-mediated cellular internalization and therapeutic efficacy may be reduced by partially obscuring targeted ligands or by surface changes intended to improve site-specific delivery [61]. Furthermore, significant interpatient heterogeneity in nanoparticle function arises from individual patient features, disease status, and plasma composition influencing protein corona composition. To reduce nonspecific protein adsorption, preserve targeting capability, and improve the reproducibility, biosafety, and clinical translation of MSN-based nanomedicines, rational surface engineering techniques such as PEGylation, zwitterionic coatings, biomimetic cell membrane coatings, and protein corona modulation are required [62].

## 4. Biomedical Applications of MSNs

MSNs have attracted considerable attention in biomedical research due to their distinctive physicochemical properties. These characteristics make MSNs potential platforms for a range of biomedical applications by facilitating the effective delivery of various therapeutic and diagnostic substances. Recent studies have demonstrated the usefulness of MSNs for targeted drug delivery—in which regulated, stimuli-responsive release systems improve therapeutic efficacy and reduce systemic toxicity [63]. Functionalized MSNs facilitate combination therapies, enhance cellular uptake, and enable targeted tumor delivery in cancer treatment. By enabling targeted delivery of antimicrobial drugs and bioactive compounds, MSN-based systems have also shown notable antibacterial and antibiofilm activity. MSNs are multipurpose carriers used in tissue engineering and regenerative medicine to transfer genes, growth factors, and regenerative therapies to promote angiogenesis, tissue repair, and cell proliferation [64]. MSNs’ uses in wound healing have expanded through their incorporation into hydrogels, scaffolds, and wound dressings. MSNs have also been extensively investigated as theragnostic and diagnostic platforms for disease monitoring, bioimaging, and personalized therapy [65].

### 4.1. Antimicrobial Applications

Triphenylphosphonium (TPP)-functionalized MSNs (TPP-MSNs) have been developed as targeted antibacterial nanocarriers, as reported in a study. TPP-MSN functionalization enhanced nanoparticle affinity for mycobacterial cells, enabling specific contact with bacterial surfaces in vitro and within infected macrophages. TPP-MSNs loaded with doxycycline showed potent antibacterial efficacy against intracellular infections, biofilms, and planktonic microorganisms. TPP-MSNs significantly reduced the bacterial burden in a zebrafish infection model and reduced bacterial uptake by host immune cells [66]. Copper-doped MSN (CuMSNs) and in situ-formed copper stearate are combined to create multifunctional cotton fabrics. While copper stearate offers instantaneous contact-killing activity and improves surface water repellency, CuMSNs enable prolonged copper-ion release via a mesoporous reservoir. Antibacterial evaluations reduced both Gram-positive and Gram-negative bacterial growth [67]. A study reported the synthesis of Ag/Zn/MCM-48 as a multifunctional antibacterial agent targeting resistant infections. Nanomaterials exhibit strong antibacterial action against a variety of multidrug-resistant bacterial strains. Ag/Zn/MCM-48 shows much lower cytotoxicity and stronger antibiofilm performance, achieving up to 90% biofilm inhibition [68]. Amine-functionalized MSNs-NH_2_-ofloxacin have been developed as an enhanced antimicrobial drug delivery system. The aminated mesoporous silica framework enables effective Ofloxacin loading, prolonged release, and increased bioavailability. Biological evaluations show markedly increased antibacterial and antibiofilm activity against clinical isolates resistant to free ofloxacin [69].

**Figure 2:**
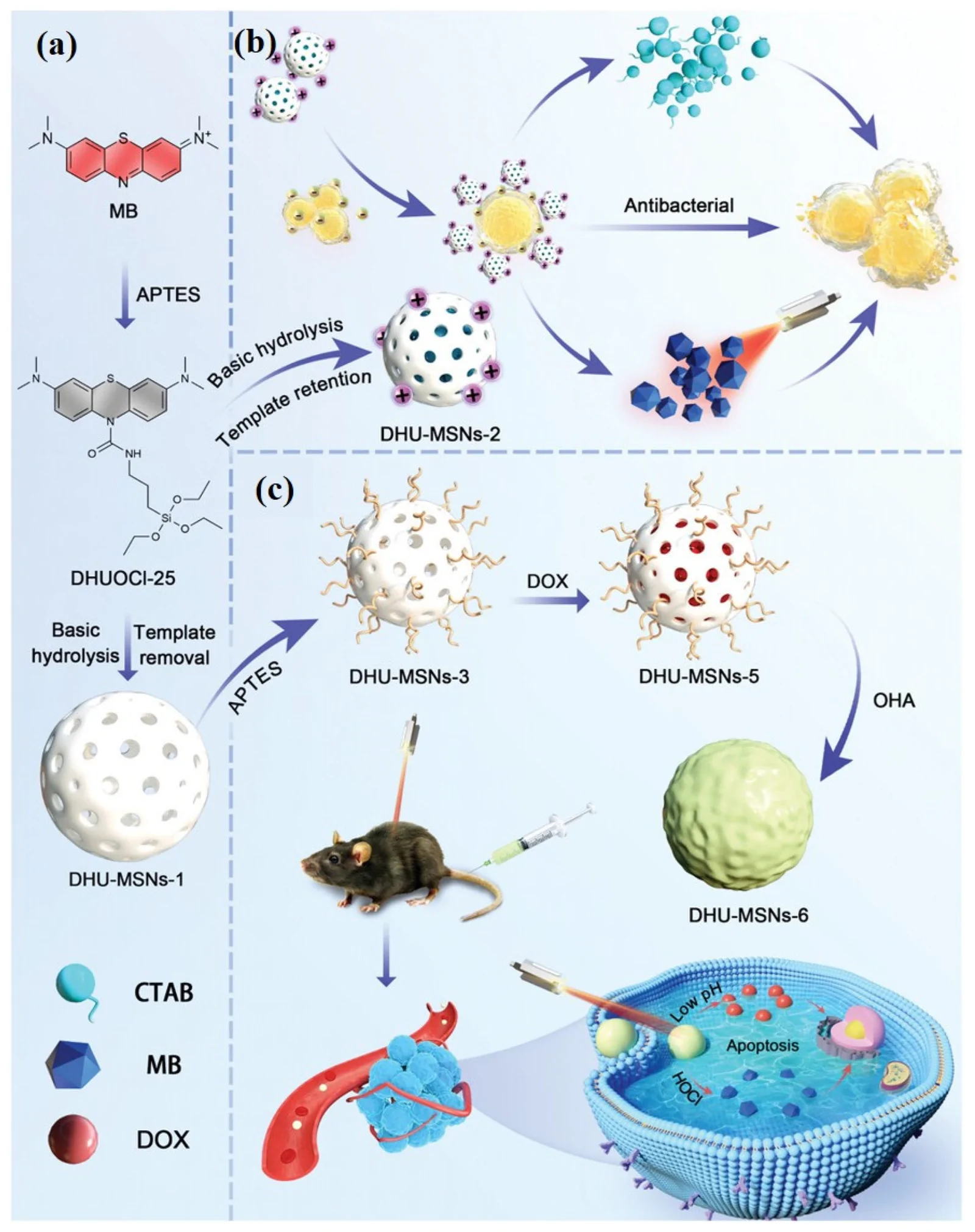
(**a**) Synthesis of aPS (DHUOCl-25) and aPS-modified MSNs (DHU–MSNs-1). (**b**) Synthesis of DHU–MSNs-2 and its photodynamic antibacterial mechanism. (**c**) Synthesis of DHU–MSNs-6 and its chemo-photodynamic therapeutic strategy. Reproduced from [70] under the CC BY 4.0 License, via Wiley Online Library.

An activatable photosensitizer (aPS)-integrated MSN platform was developed. The produced aPS, DHUOCl-25, serves as a silica precursor for synthesizing functionalized MSNs and as a hypochlorous acid-responsive photosensitizer. These nanostructures enable a variety of therapeutic applications, preserve photodynamic activity, and provide high drug-loading capacity. The resulting systems exhibit improved antimicrobial activities (Figure 2). The DHU-MSNs-2 nanoplatform exhibited potent antibacterial activity against both *S. aureus* and *E. coli*, particularly upon HOCl activation and laser irradiation. Live/dead staining and SEM analyses confirmed extensive damage to bacterial membranes, demonstrating the effectiveness of activatable photodynamic antibacterial therapy [70]. A two-step plasma-assisted method was used to functionalize the surface of Ag/MSNs-R. After uniformly depositing AgNPs onto mesoporous silica carriers and modifying them with an amine-fluorocarbon polymer layer, the resulting positive surface charge made the particles more readily electrostatically attracted to negatively charged bacterial membranes. Ag/MSNs-R exhibited significantly greater antibacterial activity than traditional Ag/MSNs, achieving over 98% reduction in both *S. aureus* and *E. coli* [71]. MSNs loaded with arginine (Arg@MSNs) were created as functional fillers to improve the base resins of 3D-printed dentures. As the concentration of nanoparticles increased, so did the antimicrobial activity against *C. albicans* and *S. mutans* [72]. MSNs loaded with dual drug conjugates for the treatment of multidrug-resistant tuberculosis (MDR-TB) have been developed. The modified nanocarriers exhibited improved bioavailability, regulated release profiles, high drug-loading capacity, and a homogeneous mesoporous architecture. DSM-loaded MSNPs exhibited greater synergistic antibacterial efficacy against *M. tuberculosis* H37Rv among the formulations studied [73]. Rutin, a biocompatible flavonoid, can serve as both a reducing agent and a nonsurfactant template in the environmentally friendly synthesis of Ag-MSNs. The Ag-MSNs demonstrated broad-spectrum antimicrobial efficacy against *E. coli* and *S. aureus* [74].

Thioketal-linked methoxy poly(ethylene glycol) (mPEG-TK)-functionalized ROS-responsive MSNs have been created as stimuli-responsive gatekeepers for regulated antibiotic delivery. Under physiological settings, vancomycin-loaded MSNs showed slower drug release. After therapy, in vivo trials demonstrated complete clearance of bacterial infection and efficient tissue recovery [75]. Quaternary ammonium chitosan-modified MSNs (MSN-NH_2_-CFP@HACC) have been developed for targeted antibiotic delivery. This nanoformulation shows strong antibacterial action against intracellular MRSA, pH-responsive drug release, and accelerated antibiotic delivery into acidic intracellular compartments. Additionally, the chitosan coating reduces antimicrobial resistance by enhancing bacterial capture, disrupting bacterial membrane integrity, and inhibiting β-lactamase activity [76]. Curcumin was used to fabricate a superparamagnetic nanocarrier loaded with ciprofloxacin (Fe-MSNPs@Cipro), which exhibited good physicochemical stability and tunable drug-release properties. Fe-MSNPs@Cipro demonstrated notable antimicrobial activity against *E. coli* and *S. aureus*, with inhibition zones of 31.33 and 23.33 mm. The corresponding MIC values were 6.09 µg/mL for *E. coli* and 12.18 µg/mL for *S. aureus*. Fe-MSNPs@Cipro exhibited greater antibacterial activity against *E. coli* compared with *S. aureus* [77]. MSN-coated plasmonic nanostructures (Cu_2−x_S@MSS) were used for the treatment of implant-associated bone infections. Cu_2−x_S triangular nanoplates form the photothermally active core of the nanosystem, which is encased in a mesoporous silica shell designed to hold antimicrobial agents such as rifampicin and levofloxacin. *S. aureus* growth has been significantly inhibited by antibacterial tests, highlighting the increased therapeutic potential of these drug-loaded nanostructures [78]. A study reported that calcium fructoborate-loaded MSN (CF@SBA-15) was used as a resin-based pulp capping material. Without adversely affecting solubility, adding CF@SBA-15 improved the material’s physicochemical characteristics by reducing water absorption. Furthermore, CF@SBA-15 enhanced biocompatibility and dramatically elevated antibacterial activity against *L. casei* and *S. mutans* [79].

The development of pectin-coated Pro@MSN-Pec as a stimuli-responsive fungicide delivery system has been described. This nanoformulation exhibits effective drug loading and pectinase-induced enzyme-responsive release. Fluorescence-tracking investigations confirm effective nanoparticle absorption, transport, and distribution throughout rice plant tissues. Pro@MSN-Pec outperforms in plant uptake, prolonged retention, and enhanced antifungal efficacy against *M. oryzae* [80]. A study reported the development of asymmetric lollipop-shaped Fe_3_O_4_@SiO_2_&EPMO with dual-compartment designs featuring separate hydrophilic and hydrophobic domains, enabling simultaneous loading of multiple medicinal compounds. A spherical Fe_3_O_4_@SiO_2_ head and an ethane-bridged periodic mesoporous organosilicon nanorod tail constitute these anisotropic nanoparticles, which provide independent storage spaces and a large surface area for dual-drug encapsulation. In an in vitro study, these nanoparticles were more toxic to cancer cells than free pharmaceuticals, and dual-drug-loaded nanoparticles also showed strong antibacterial activity [81]. The creation of resin using amino-silane-functionalized zinc oxide quantum dots and pore-expanded MSNs loaded with chlorhexidine. Even after a month of aging, the improved formulation retained long-term antibiofilm effectiveness against *S. mutans* and sustained chlorhexidine release for up to 30 days [82].

Amine-functionalized ZnFe_2_O_4_@MS has been reported as a magnetic nanoplatform for immobilizing Candida rugosa lipase. The immobilized lipase demonstrates improved thermal stability, reusability, and magnetic recoverability, and continues to exhibit notable activity even after several operational cycles. Additionally, the ZnFe_2_O_4_@MS exhibits antibacterial activity, particularly against *S. aureus* [83]. Silver-loaded MSNs were incorporated into a stable, compact nanocomposite coating for controlled antibacterial applications. Silver ions were efficiently absorbed by amino-functionalized MCM-41 nanoparticles, which subsequently transformed them into evenly distributed silver nanoparticles inside the mesoporous matrix. The resulting nanocomposite coating exhibited consistent nanoparticle distribution, preserved structural integrity, and enabled prolonged ion release. Prolonged biocidal activity was supported by controlled-release tests showing the slow diffusion of antibacterial agents [84]. Zinc-doped, carboxyl-functionalized MSNs (MSN@Zn-COOH) have been developed as multifunctional agents. While carboxyl surface functionalization enhances blood compatibility and hemostatic efficiency, adding zinc ions provides potent antibacterial effects against *E. coli* and *S. aureus*. Rapid coagulation, reduced activated partial thromboplastin time, low hemolytic activity, and significant cytocompatibility were observed [85].

**Figure 3:**
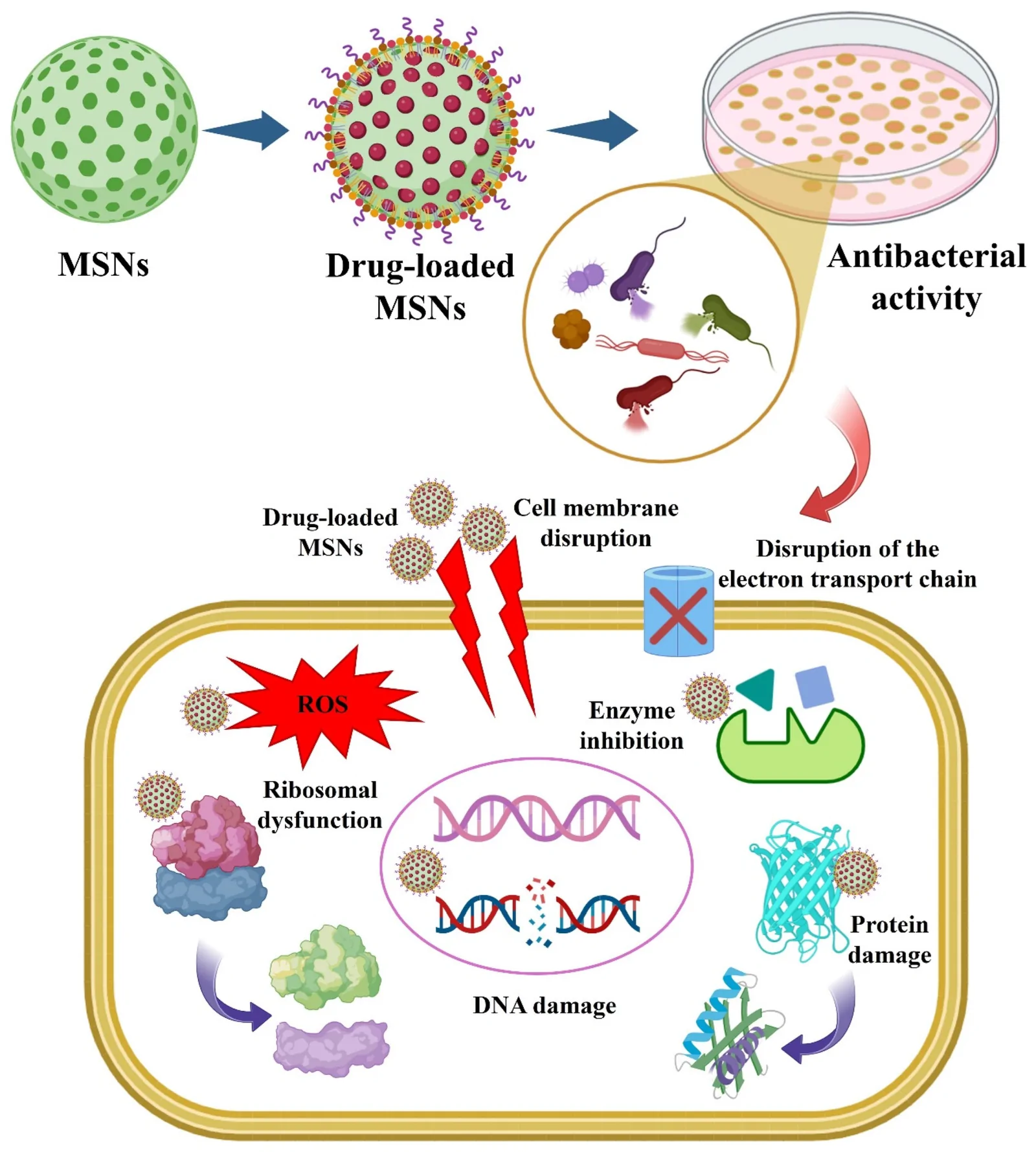
Schematic illustration of the antibacterial mechanism of drug-loaded MSNs. After interacting with the bacterial cell surface, drug-loaded MSNs disrupt the bacterial cell membrane, generate ROS, inhibit enzymes, disrupt the electron transport chain, damage DNA, impair ribosomal function, and denature proteins, ultimately inhibiting bacterial growth and causing cell death (created using BioRender.com).

Drug-loaded MSNs demonstrate potent antibacterial activity through several complementary mechanisms that collectively enhance bacterial eradication and reduce the risk of antimicrobial resistance. When MSNs contact bacterial surfaces, they enable localized, sustained release of encapsulated antimicrobial compounds that compromise cell membrane integrity and increase membrane permeability [86]. After intracellular uptake of the delivered therapies, excess ROS are produced, leading to oxidative stress, lipid peroxidation, and damage to vital cellular components. Drug-loaded MSNs simultaneously affect ATP synthesis and energy homeostasis by inhibiting key enzymes and disrupting the electron transport chain, thereby disrupting bacterial metabolism. Furthermore, these nanoplatforms hinder bacterial proliferation and cellular repair by inducing irreversible DNA and protein damage and inhibiting protein synthesis through ribosomal malfunction [87]. Broad-spectrum antibacterial efficacy against both Gram-positive and Gram-negative bacteria is achieved by combining controlled drug release, ROS-mediated oxidative damage, membrane rupture, metabolic inhibition, and macromolecular damage [88]. By improving therapeutic efficacy and reducing systemic toxicity, these multifunctional mechanisms make drug-loaded MSNs highly promising nanocarriers for treating biofilm-associated, chronic wound, and multidrug-resistant bacterial infections (Figure 3).

A study describes the development of amine-functionalized MSNs loaded with piperacillin (MSN-NH_2_-PIP) as a nanotherapeutic platform. This nanoformulation enables regulated, pH-responsive drug release, improving antibiotic delivery in acidic environments. Against clinically relevant MDR pathogens, MSN-NH_2_-PIP shows much greater antibacterial activity than free piperacillin. Mechanistic studies show that the formulation exhibits potent antibiofilm activity, induces oxidative stress, and damages bacterial membranes [89]. Plant-derived medicinal compounds have been encapsulated in mesoporous silica-based carriers, namely MCM-41 and MCM-48 nanoparticles, both with and without amino-functionalization. Physicochemical studies confirmed successful incorporation of bioactive polyphenols into the mesoporous matrices. Significant activity against methicillin-resistant *S. aureus* was observed, and bacterial virulence factor synthesis was inhibited [90]. An MSN-based antimicrobial nanodevice co-loaded with curcumin and polymyxin B was developed. Curcumin produced ROS when exposed to blue light, and polymyxin B achieved a higher level of bacterial eradication than the free agents. This nanoformulation dramatically reduced biofilm formation and demonstrated significant antibacterial activity against *P. aeruginosa*, *S. epidermidis*, and *E. coli* [91]. MSNs co-loaded with lysozyme and vancomycin have been developed as a combined antibacterial nanotherapeutic platform. By enabling the simultaneous delivery of both antimicrobial drugs, this MSN-based carrier creates a synergistic antibacterial effect against *S. aureus*. Compared with their free forms, the nanoformulation significantly reduces the minimum inhibitory concentrations of lysozyme and vancomycin, indicating improved antibacterial efficacy and lower dose requirements [92].

Fe_3_O_4_@mSiO_2_@CysHHC10 has been reported as a robust and recyclable antibacterial platform. Fe–S bond interactions stabilize the antimicrobial peptide CysHHC10 inside this nanostructure, which has a mesoporous silica shell covering a magnetic Fe_3_O_4_ core. The resultant nanocomposite exhibits strong antibacterial activity against various bacteria [93]. The encapsulation of thyme essential oil (TEO) into dendritic MSNs (DMSNs) has been shown to enhance antifungal activity against *F. oxysporum*. Compared with free TEO, the dendritic pore structure enabled effective loading and prolonged TEO release. TEO-loaded DMSNs demonstrated a dose-dependent suppression of fungal growth of up to 70%. Mechanistic studies revealed that the nanocomposite damaged the integrity of the fungal cell membrane, allowing internal contents to seep out and ultimately leading to cell death [94]. A multifunctional titanium implant (Ti-M@A) functionalized with diselenide-bridged MSNs, loaded with the antimicrobial peptide HHC36, is described in a study. By rupturing bacterial membranes and preventing biofilm formation, the modified implant demonstrated strong antibacterial activity (>95%) against a range of clinically significant infections [95]. Mesoporous silica-coated AgNPs (Ag@mSiO_2_) have shown substantial antibacterial activity against various bacteria. The size of the silver core affects Ag@mSiO_2_′s antibacterial effectiveness; amine functionalization further improves it by adding a positive surface charge. Polyurethane films containing Ag@mSiO_2_ exhibit enhanced bactericidal efficacy [96]. A citric acid-assisted approach was used to sustainably synthesize MSN from rice hull waste, yielding materials with superior porosity compared to those produced by traditional incineration. The green production of silver-doped MSN, which showed strong antibacterial activity, was subsequently carried out using the extracted MSN. The results showed uniform nanoparticle distribution and notable improvements in antibacterial performance, UV protection, and color properties [97].

*Angelica gigas* Nakai extract served as a green reducing agent to produce mesoporous silica-coated multi-walled carbon nanotubes (MWCNTs) adorned with AgNPs. The mesoporous silica coating enabled homogeneous AgNPs deposition within the porous matrix and enhanced MWCNT dispersibility. As antimicrobial efficiency increased in direct proportion to AgNPs loading, the resulting nanocomposite exhibited strong antibacterial activity against both *S. aureus* and *E. coli*. Furthermore, the nanomaterial prevented bacterial biofilm formation, a key contributor to antibiotic resistance [98]. MSNs functionalized with ε-poly-L-lysine and loaded with cinnamaldehyde were used to create a stimuli-responsive antibacterial nanodevice. Through the enzymatic hydrolysis of the polypeptide cap by proteolytic enzymes released by pathogenic microbes, this technology enables the biocontrolled release of cinnamaldehyde. Compared with free cinnamaldehyde, it demonstrated noticeably higher antibacterial activity against *E. coli*, *S. aureus*, and *C. albicans* [99]. A study showed that hollow magnetic mesoporous silica spheres exhibit strong antibacterial and drug-delivery properties. The hollow core, porous shell, high surface-to-volume ratio, and magnetic responsiveness enhance these nanostructures’ potential for biomedical applications. Antibacterial testing showed significant activity against several harmful bacteria, including *A. baumannii* [100]. A study developed an MSN-based nanophotosensitizers incorporating a hydrophobic squaraine dye (Br-SQ) has been described. By improving photosensitizer dispersion and delivery, the MSN platform addresses the poor solubility and aggregation of squaraine dyes. A thorough physicochemical analysis confirms that Br-SQ was successfully incorporated into the mesoporous structure. The nanophotosensitizers show strong antibacterial activity against *S. aureus* and *E. coli* under visible light at 640 nm [101].

Although MSN-based antibacterial, antifungal, and stimuli-responsive drug delivery systems have demonstrated remarkable therapeutic efficacy in experimental studies, their performance varies considerably between planktonic bacteria and mature biofilms, which represent distinct therapeutic challenges. Planktonic bacteria are generally more susceptible to antimicrobial agents because of their high metabolic activity and direct exposure to antibiotics [102]. In contrast, mature biofilms consist of densely organized microbial communities embedded within a protective extracellular polymeric substance (EPS) matrix that restricts nanoparticle penetration, limits drug diffusion, promotes bacterial persistence, and facilitates multidrug resistance. Consequently, effective biofilm eradication requires multifunctional strategies that can penetrate the EPS matrix, disrupt biofilm architecture, generate ROS, deliver high local concentrations of antimicrobial agents, eliminate dormant bacterial populations, and prevent biofilm recurrence [103]. Although tannic acid and tetracycline-co-encapsulated MSNs have shown significant potential through stimuli-responsive drug release, ROS-mediated antimicrobial activity, membrane disruption, and localized drug delivery, extracellular matrix barriers, and disease-specific microenvironments. Furthermore, enzyme activity, and ROS concentrations can affect the predictability and consistency of stimulus-responsive drug release [104]. Therefore, future investigations should use clinically relevant chronic wound and implant-associated infection models that incorporate mature biofilms, along with standardized pharmacokinetic, biodistribution, and long-term efficacy evaluations, better to assess the translational potential of MSN-based antimicrobial nanotherapeutics.

Despite encouraging results in murine infection and tumor models, most MSN-based antibacterial and antifungal studies remain limited to short-term efficacy evaluations and provide insufficient information regarding the pharmacokinetics (PK), organ biodistribution, biodegradation, and long-term biological fate of these nanoplatforms. Comprehensive quantitative analyses of nanoparticle absorption, circulation time, tissue penetration, metabolism, degradation kinetics, and clearance pathways are still lacking [105]. In particular, the influence of particle size, pore architecture, surface functionalization, and protein corona formation on organ-specific accumulation within the liver, spleen, lungs, kidneys, and infected tissues following repeated administration remains poorly understood. Furthermore, few studies have systematically investigated long-term tissue retention, orthosilicic acid clearance, chronic inflammation, immunotoxicity, fibrosis, or organ-specific toxicity after repeated dosing [106]. The lack of standardized pharmacokinetic protocols and clinically relevant animal models further limits cross-study comparisons and hinders regulatory evaluation. Therefore, future research should prioritize comprehensive PK profiling, quantitative biodistribution analyses, real-time molecular imaging, repeated-dose toxicity studies, long-term biodegradation assessments, and standardized preclinical evaluation to establish the safety, efficacy, and clinical translatability of MSN-based antimicrobial nanotherapeutics. Table 1 highlights the rapid development of MSN-based antibacterial platforms with diverse surface modifications, antimicrobial cargos, and controlled drug-delivery strategies to combat bacterial infections. Notably, surface-functionalized MSNs exhibited enhanced bacterial targeting, sustained antimicrobial release, and improved biofilm eradication against a broad spectrum of pathogenic microorganisms. Collectively, these findings underscore the importance of optimizing MSN design and antibacterial efficacy to facilitate clinical translation.

### 4.2. Cancer Theragnostics and Therapy

Smart MSNs have emerged as versatile nanoplatforms for cancer theragnostics due to their physicochemical properties. Their stimuli-responsive behavior enables controlled drug release in the tumor microenvironment while simultaneously supporting diagnostic imaging for real-time treatment monitoring. These multifunctional properties improve therapeutic efficacy, enhance targeting specificity, and minimize systemic toxicity, making smart MSNs promising candidates for precision oncology [108]. The development of chitosan-functionalized MSNs co-loaded with chrysin and quercetin (Chr–Qur@MSNs–Chi) has been described. With particle sizes ranging from about 80 to 110 nm and a strongly negative surface charge, the nanosystem exhibits a spherical morphology that confers robust colloidal stability. The system showed strong cytotoxicity in vitro against A549 lung cancer cells and greater efficacy than free-drug combinations. The nanocarrier significantly induces apoptosis and G0/G1 cell cycle arrest, as evidenced by downregulation of proliferative and anti-apoptotic markers (Bcl-2, Cyclin D1, pRB) and upregulation of pro-apoptotic genes (p53, Bax, Fas) [109]. Hyaluronic acid-modified MSNs loaded with cisplatin (HA-MSN-CDDP) were developed to target CD44-overexpressing lung cancer cells through HA-mediated receptor-dependent endocytosis. Compared with free cisplatin, HA-MSN-CDDP induced greater apoptosis in A549-Luc-C8 lung cancer cells while reducing cytotoxicity, genotoxicity, and oxidative stress in healthy CCD-34Lu fibroblast cells. In vivo, HA-MSN-CDDP effectively suppressed tumor growth, reduced lipid peroxidation, and preserved antioxidant enzyme activities [110]. The green synthesis of aminopropyltriethoxysilane-functionalized MSNs-AgNPs utilized pomegranate extracts as natural reducing agents. These nanocarriers have good physicochemical properties, are highly biocompatible, and efficiently load doxorubicin. Controlled-release experiments verify that the MSN-based system can efficiently deliver doxorubicin to cancer cells. In vitro studies show increased cytotoxicity against cervical and breast cancer cells while preserving compatibility with normal fibroblast cells [111].

Dendritic MSNs combined with Fe^2+^ ions, glucose oxidase (GOx), and a polydopamine (PDA) coating were used to create a multifunctional nanoplatform (GOx@Fe-DMSN@PDA, GFDP) for synergistic cancer treatment. For PTT, the PDA shell enabled efficient photothermal conversion and pH- and NIR-responsive controlled release. Under NIR irradiation, in vitro experiments showed strong synergistic anticancer effects and a marked decrease in cell viability compared with non-irradiated controls. An in vivo study confirmed effective tumor accumulation and improved treatment efficacy [112]. MSNs-ABC@PDA-OVA is a multifunctional MSNs-based nanovaccine that combines ovalbumin, polydopamine, and ammonium bicarbonate for synergistic photothermal-immunotherapy of melanoma. MSNs-ABC@PDA-OVA shows strong photothermal properties and enables laser-triggered antigen release and endosomal escape, enhancing dendritic cell activation, maturation, and migration to tumor-draining lymph nodes. Consequently, the nanovaccine creates long-lasting immunological memory, eliminates initial tumors, and elicits strong anticancer immune responses (Figure S1a) [113]. A study developed angiopep-2-functionalized liposome–mesoporous silica hybrid nanoparticles (ANG-LP-PAA-MSN) for targeted glioma therapy. This nanoplatform effectively delivers arsenic trioxide to glioma tissues by combining drug release with enhanced penetration of the blood–brain barrier (BBB). In vitro, it showed improved cellular uptake, transport efficiency, and apoptotic induction. In vivo pharmacokinetic assessments revealed prolonged circulation time and markedly improved tumor-targeting efficacy. ANG-LP-PAA-MSN@ATO showed improved therapeutic efficacy in intracranial glioma-bearing rats and increased accumulation in glioma tissues, highlighting its potential as an advanced nanocarrier system for targeted brain cancer treatment (Figure S1b) [114].

A multifunctional mesoporous silica-based nanoplatform (MSNCDs-FA@TMPyP) has been developed for combined chemo–photodynamic cancer therapy. Thorough physicochemical characterization validated successful synthesis, spherical morphology (100–200 nm), and efficient surface functionalization. In vitro experiments with MDA-MB-231 breast cancer cells showed cytotoxicity upon light activation, accompanied by a notable increase in the apoptotic cell population. Mechanistically, the platform integrated ROS-mediated cell death, controlled release, and targeted delivery to enable synergistic photodynamic and chemotherapeutic effects [115]. A study details the development of a hybrid nanocarrier lipid-coated MSNs to improve doxorubicin delivery. Nanoscale dimensions of about 98 nm, a large pore capacity, and effective structural alteration. Compared with the free drug and non-targeted formulations, folate-functionalized, doxorubicin-loaded nanoparticles exhibited significantly higher cytotoxicity and improved cellular absorption in vitro. Additionally, lipid-coated, folate-modified MSNs reduced doxorubicin-induced cardiotoxicity in H9c2 cells, suggesting improved safety [116]. An enzyme-responsive MSN system for targeted drug delivery in hypoxic tumor settings has been developed. The Azo cap stays stable and stops early drug release in normoxic conditions. In contrast, overexpressed azoreductase cleaves the gatekeeper under hypoxic conditions, enabling regulated doxorubicin release. In vitro experiments comparing hypoxic, azoreductase-rich A549 cells with low-enzyme-expressing THP-1 cells show improved drug release and markedly increased cytotoxicity [117].

MSNs containing phosphonate groups and coated with folic acid, chitosan, and glycine proved to be an effective targeted drug-delivery system for colchicine (COL). With this improved nanoformulation, COL was substantially less harmful to normal BJ1 cells and more effective against HCT116, HepG2, and PC3 cancer cells. Subsequent research showed that by increasing antimitotic activity and modulating key molecular targets such as MALAT1, miR-205, Ang-2, and PD-1, the formulation induced intrinsic apoptosis [118]. A study described the development of dual-responsive, alginate-coated MSNs designed for oral co-delivery of curcumin and quercetin. This nanohybrid exhibited a homogeneous particle size below 50 nm and a significant polyphenol-loading capacity. Combining the alginate coating with a disulfide-linked structure improved the distribution of encapsulated polyphenols in the intestinal and colonic environments, thereby enabling stimuli-responsive release. Because of its enhanced stability, higher bioavailability, and synergistic therapeutic effects, the nanoformulation demonstrated noticeably greater anticancer efficacy against HT29 colon cancer cells than free curcumin and quercetin [119]. Umbelliprenin-loaded polydopamine-coated MSNs (UMB-MSN-PDA) have been created as a sustained-release anticancer delivery method. This nanoformulation demonstrates high drug encapsulation efficiency, advantageous physicochemical properties, and prolonged drug release. There is reduced toxicity to normal human fibroblasts and specific cytotoxicity against MCF-7 breast cancer cells. Mechanistic studies show that UMB-MSN-PDA increases the number of apoptotic cells by upregulating key apoptotic markers, including p53, caspase-8, and caspase-9 [120].

Curcumin and the photosensitizer IR780 have been co-loaded into polyethylene glycol-capped MSNs (Cur&IR780@MSN) for combined chemotherapy aimed at lung cancer. This versatile nanocarrier demonstrates effective drug encapsulation, curcumin release, enhanced reactive oxygen species generation under near-infrared irradiation, and biocompatibility. In vitro, the combination treatment shows superior anticancer activity against A549 lung cancer cells and greatly outperforms individual therapies, reducing cell viability by up to 78%. Additionally, the nanoformulation prevents cancer cell migration, indicating improved therapeutic potential [121]. The development of phospholipid-coated MSNs for the transport of piperlongumine, a naturally occurring compound with anticancer properties, has been described. The MSN lipid bilayer exhibited improved pH responsiveness and sustained drug release. Under acidic conditions typical of the tumor microenvironment, the lipid-coated formulation exhibited enhanced release and prolonged drug retention. Furthermore, compared with non-coated nanoparticles, silicasomes exhibited dramatically enhanced cytotoxicity and cell death in MCF-7 breast cancer cells [122]. To overcome drug resistance in melanoma therapy, paclitaxel-loaded MSNs were co-administered with curcumin and D-α-tocopherol polyethylene glycol 1000 succinate (TPGS). Curcumin and TPGS prevent P-glycoprotein-mediated drug efflux and increase intracellular drug accumulation, while MSNs enhance paclitaxel solubility and delivery. The PS+CUR+TPGS combination exhibits greater anticancer activity than free paclitaxel, significantly increasing apoptosis and microtubule disruption. In vivo studies demonstrate a significant reduction in tumor growth and selective accumulation of paclitaxel within tumor tissues without increasing systemic exposure [123]. Under mildly acidic, tumor-like target conditions, polyacrylic acid-functionalized MSNs exhibit reversible, pH-dependent swelling and shrinking, thereby enabling controlled drug release. Because of the synergistic anticancer effects of the combined chemotherapeutics, the dual-drug formulation (CP/DOX@MSNs-PAA) showed superior therapeutic efficacy compared with single-drug systems. The dual-loaded nanocarriers greatly enhanced cytotoxicity toward cancer cells [124].

Dual drug-loaded PLG/GEM@MSNPs/MTX@MSNPs have been developed for the combination therapy of pancreatic ductal adenocarcinoma. Compared with single-drug formulations, PLG/GEM@MSNPs/MTX@MSNPs exhibited improved antiproliferative activity against PANC-1 pancreatic cancer cells [125]. An MSN coated with a lactobionic acid-based glycopolymer (MSN-glycopolymer) has been developed for the targeted co-delivery of nucleic acids. MSN-glycopolymer nanosystems exhibit strong physicochemical properties and dramatically improved gene-delivery performance. Furthermore, by combining HSV-TK/GCV suicide gene therapy with epirubicin chemotherapy, the platform facilitates a synergistic treatment strategy that increases anticancer activity [126]. A study developed a tumor microenvironment-responsive copper peroxide–mesoporous organosilica core–shell nanoplatform for targeted ferroptosis-enhanced chemotherapy. These ROS-produced materials in the tumor microenvironment induce ferroptosis and enhance chemotherapy efficacy. In vitro tests show that Apt-PEG-Silica-DOT@DOX outperforms the non-targeted PEG-Silica-DOT@DOX formulation in terms of cellular uptake and cytotoxicity. While maintaining a good biosafety profile, Apt-PEG-Silica-DOT@DOX performs considerably better in vivo, achieving over 90% tumor suppression [127].

**Figure 4:**
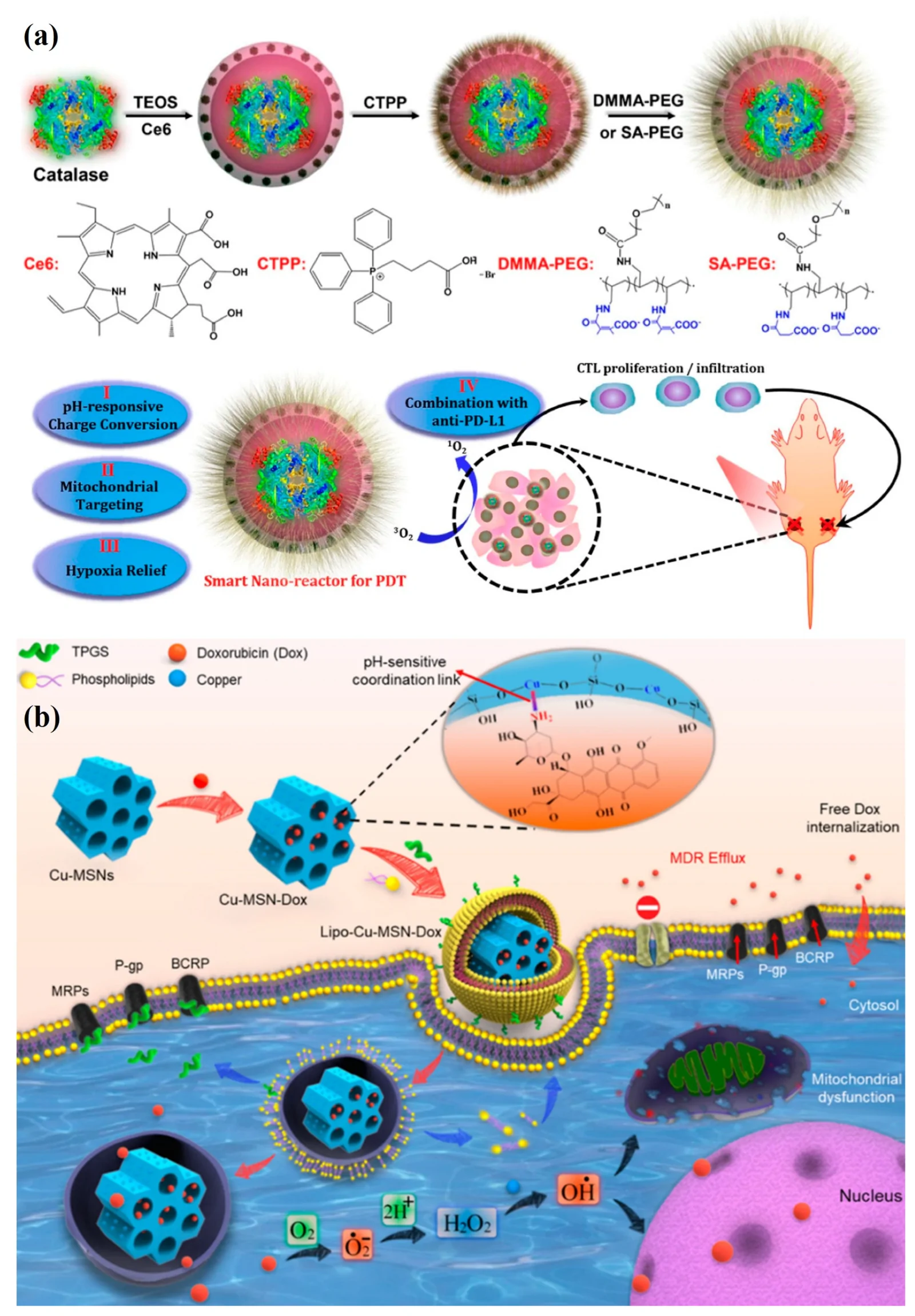
(**a**) Schematic illustration of the multistage-responsive silica nanoparticle system incorporating catalase, chlorin e6, mitochondria-targeting ligands, and pH-responsive polymers for enhanced photodynamic therapy. Reproduced from [128] under the CC BY 4.0 License, via ACS. (**b**) The TPGS-coated Cu-MSN nanoplatform for pH-responsive doxorubicin delivery and multidrug resistance reversal. The Lipo-Cu-MSN-Dox system enhances intracellular drug accumulation and induces ROS-mediated mitochondrial apoptosis through copper-triggered Fenton-like reactions. Reproduced with permission from [129] under the CCC License, ACS.

A multistage-responsive silica nanoparticle platform was developed to overcome inadequate tumor targeting and hypoxia-induced resistance in photodynamic therapy (PDT). The platform incorporates mitochondrial-targeting ligands, surface coatings for enhanced tumor homing and intracellular delivery, catalase (CAT) for oxygen generation, and chlorin e6 (Ce6) as a photosensitizer. CAT-mediated decomposition of endogenous H_2_O_2_ alleviated tumor hypoxia and enhanced ROS generation during PDT. Moreover, combining nanoparticle-mediated PDT with immune checkpoint blockade promoted systemic antitumor immunity and inhibited both primary and distant tumor growth (Figure 4a) [128]. A hierarchical copper-substituted MSNs (Cu-MSN) system was developed to address multidrug resistance (MDR) in cancer therapy. Through pH-sensitive coordination bonds, doxorubicin (Dox) was bound to the Cu-MSN-Dox framework, enabling regulated drug release in the acidic tumor microenvironment. To improve intracellular drug retention and prevent P-glycoprotein-mediated drug efflux, TPGS-containing liposomes were applied to the nanoparticles. Additionally, copper ions induced ROS formation via Fenton-like processes, causing apoptosis and mitochondrial dysfunction in resistant cancer cells. This multipurpose nanoplatform greatly increased cytotoxicity and has great potential to overcome MDR in cancer therapy (Figure 4b) [129].

Enzyme-functionalized MSN systems can improve tumor penetration by degrading the ECM. Through electrostatic interactions and pore confinement, cationic MSNs with tunable pore diameters immobilized proteolytic enzymes such as hyaluronidase, bromelain, and collagenase. Enzyme conformation, surface charge, and pore structure all contributed to the high enzyme-loading capacity and regulated release of the optimized formulations. Among these systems, collagenase-loaded CMSNs penetrated the thick extracellular matrix of pancreatic tumors most successfully. The enzyme–MSN complexes exhibited greatly increased anticancer activity when loaded with doxorubicin, resulting in potent in vivo suppression of tumor growth [130]. In a breast cancer mouse model, tin-conjugated MSNs (MSN-SnPh3; S3) showed strong in vivo anticancer efficacy and safety. By increasing ROS, promoting apoptosis, and reducing Ki-67 expression, S3 therapy dramatically inhibited tumor development. Toxicological assessments revealed no appreciable alterations in organ histology, metabolic processes, or serum biochemical markers [131]. Lactobionic acid-mediated LA-CMCS-MSN@CUR has been developed a study for targeted curcumin delivery against hepatocellular cancer. Through effective drug loading, drug release, and liver cancer cell-specific targeting via LA receptor-mediated endocytosis, this nanocarrier increases curcumin bioavailability. In vivo studies using orthotopic and solid-tumor liver cancer mouse models show greater anticancer efficacy. Mechanistically, the therapy promotes apoptosis by increasing caspase-3 and caspase-8 activation, while suppressing tumor angiogenesis and survival pathways by downregulating VEGF, p-PI3K, and AKT expression [132]. A study created MSNs functionalized with folic acid–chitosan (FA–CS) for targeted administration of breast cancer treatment. While keeping the medication in an amorphous state to enhance water solubility, FA-CS surface modification dramatically boosted PTL loading and entrapment efficiency. The nanocarrier demonstrated diffusion-controlled drug release, with greater PTL release under acidic, tumor-like conditions than at normal pH. Folate receptor-mediated endocytosis promoted cellular uptake, cytotoxicity, and cell death in in vitro experiments using MCF-7 breast cancer cells [133].

**Figure 5:**
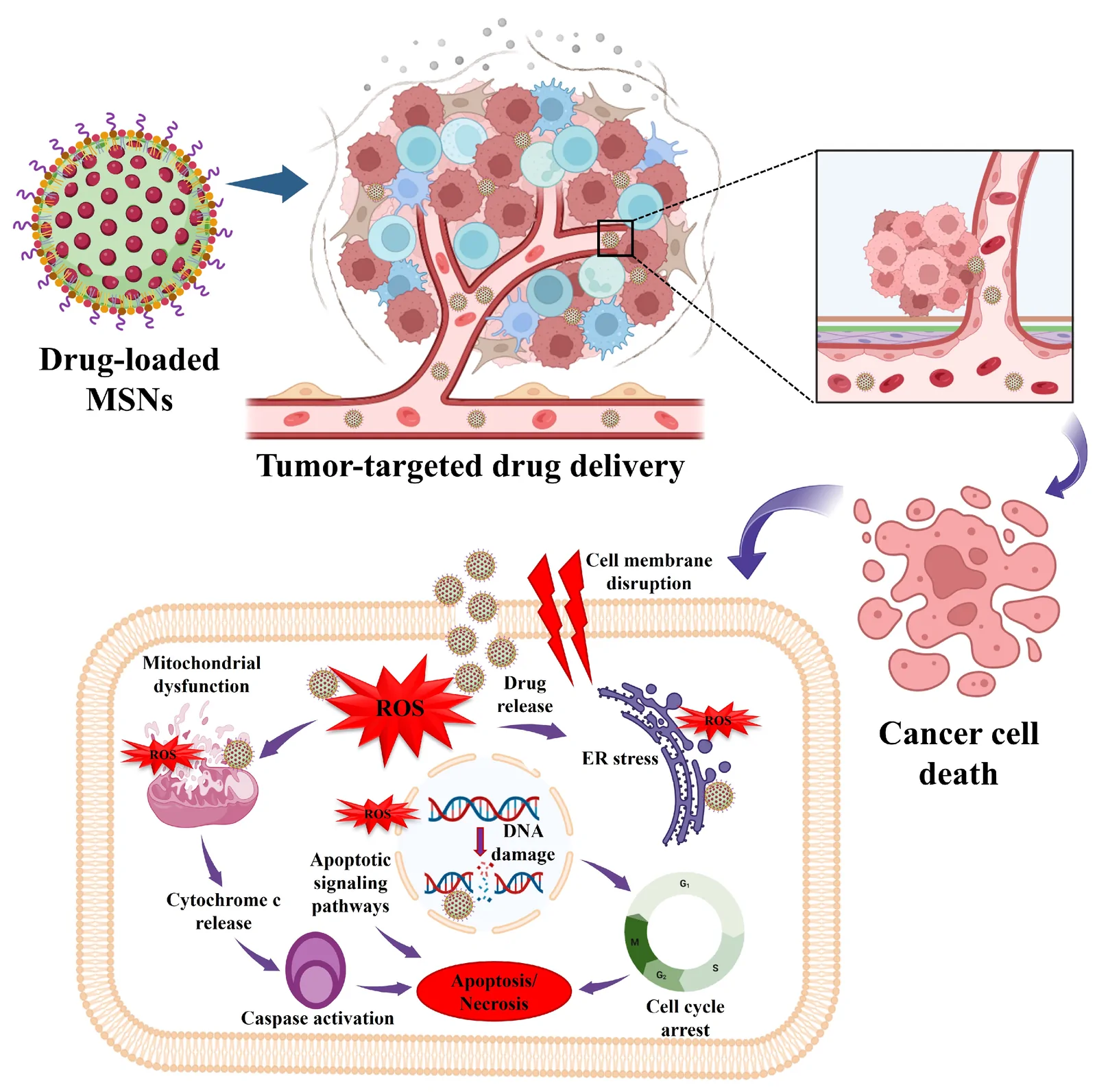
Schematic illustration of the tumor-targeted therapeutic mechanism of drug-loaded MSNs. Following tumor accumulation and cellular internalization, MSNs release their therapeutic payload, inducing ROS-mediated mitochondrial dysfunction, ER stress, DNA damage, apoptotic signaling, caspase activation, and cell cycle arrest. These coordinated intracellular events ultimately lead to apoptosis and necrosis, resulting in effective cancer cell death (created using BioRender.com).

Drug-loaded MSNs have emerged as effective nanocarriers for targeted cancer therapy due to their high drug-loading capacity, controlled release profiles, and adaptable surface functionalization. Functionalized MSNs accumulate in tumor locations by passive and active targeting following systemic treatment, allowing for localized and sustained drug release while lowering systemic toxicity [134]. Upon cellular uptake, therapeutic drugs trigger harmful mechanisms, including increased ROS formation, mitochondrial dysfunction, endoplasmic reticulum (ER) stress, and reduced cell membrane integrity. Increased intracellular ROS triggers apoptotic signaling, oxidative DNA damage, and cytochrome c release from mitochondria, leading to caspase activation and programmed cell death [135]. Concurrently, oxidative stress and DNA damage impede cell-cycle progression, leading to cell-cycle arrest and preventing tumor cell growth. Apoptosis and, in some cases, necrotic cell death result from the interplay among mitochondrial dysfunction, ER stress, oxidative damage, and activation of apoptotic pathways [136]. These integrated mechanisms highlight the potential of MSN-based drug delivery systems as targeted nanoplatforms for theragnostic and precision cancer therapy, while also improving their therapeutic efficacy (Figure 5).

Folic acid-functionalized, amine-modified MSNs (MSN-NH2) have been developed as a targeted carrier. Drug-release experiments support longer drug retention and delivery, showing a slower release rate in acidic environments. Compared with traditional therapies, cellular tests demonstrate improved biocompatibility and reduced toxicity against ovarian and cervical cancer cells. Folic acid functionalization enables receptor-mediated targeting of cancer cells that overexpress folate receptors [137]. A study describes the development of folic acid-modified, bovine serum albumin-coated hollow MSNs (HFB) as a stimuli-responsive platform for combined chemotherapy and photodynamic therapy in gastric cancer. Paclitaxel and indocyanine green were co-loaded into this nanocarrier to provide targeted delivery and complementary therapeutic benefits. The albumin coating served as a gatekeeper, enabling pH- and redox-responsive drug release within the tumor microenvironment. In contrast, folic acid promoted receptor-mediated uptake by folate receptor-overexpressing cancer cells. In vitro, high chemo-photodynamic effects, increased apoptosis, and improved cellular internalization were observed, with a strong synergistic interaction [138]. Chlorotoxin-functionalized MSNs for targeted, pH-responsive delivery of paclitaxel to treat GBM. Paclitaxel-loaded mesopores capped with β-cyclodextrin via acid-labile hydrazone linkages are incorporated into the nanocarrier. Surface modification with the glioma-targeting peptide chlorotoxin increases the delivery system’s selectivity for GBM cells via host-guest interactions. Chlorotoxin-modified nanoparticles exhibit higher cytotoxicity despite lower cellular uptake in in vitro assays with U87 glioblastoma cells, indicating greater therapeutic efficacy [139].

A study developed syndecan-1 (SDC1)-modified, lipid bilayer-coated MSNs co-loaded with gemcitabine and honokiol (SDC1-LB-MSN-GEM/HNK) for targeted pancreatic cancer therapy. This nanoplatform exhibited greater cytotoxicity than non-targeted formulations by achieving high drug encapsulation and improved cellular absorption via SDC1/IGF1R-mediated endocytosis. Mechanistic research revealed that SDC1-LB-MSN-HNK induced apoptosis via the mitochondrial route. In BxPC-3 tumor-bearing mice, SDC1-LB-MSN-GEM/HNK suppressed tumor growth by 56.19% in vivo, outperforming both free GEM/HNK and LB-MSN-GEM/HNK [140]. Docetaxel-loaded octyl-functionalized PEGylated MSN (DTX@C8-MSN) has been developed as a targeted therapy strategy for temozolomide-resistant glioblastoma. This nanocarrier exhibits enhanced drug-transport efficiency and low hemolytic activity. In vivo imaging confirms preferential accumulation of C8-MSN in glioma tissues, indicating improved tumor targeting and successful BBB penetration. When DTX@C8-MSN is administered to orthotopic glioblastoma mouse models, it significantly reduces tumor burden, increases survival by more than 50%, and decreases systemic toxicity compared with free docetaxel. Mechanistic studies show that cleaved caspase-3 and PARP signaling pathways are activated, leading to enhanced apoptosis [141]. Hyaluronic acid-coated MSNs (HA-MSNs) were synthesized for the co-delivery of selenium and doxorubicin as a combined treatment for osteosarcoma. The dual-drug delivery strategy attempts to improve doxorubicin’s anticancer effectiveness while resolving issues with nonspecific toxicity and drug resistance. According to in vitro study, osteosarcoma cell growth is considerably more inhibited by Se/Dox-laden HA-MSNs than by nanoparticles loaded with either selenium or doxorubicin alone [142]. Researchers reported a tumor microenvironment-responsive hollow MSN system (HMSN-DHA@TA/Fe) for targeted cancer treatment. Encapsulated dihydroartemisinin (DHA) in hollow mesoporous silica nanoparticles and then coated them with a tannic acid/iron complex, which acts as a pH-sensitive gatekeeper, creating this nanocarrier. This significantly decreased tumor cell survival while maintaining high viability in normal cells. Considerable tumor growth inhibition was observed in vivo, highlighting HMSN-DHA@TA/Fe’s promise as a mesoporous silica-based nanoplatform for targeted solid-tumor therapy that is both safe and effective [143].

A dual surfactant-assisted approach was used to create CSMS in a study. The produced nanoparticles demonstrate biocompatibility and controlled drug release, with enhanced release in acidic environments. Multiple analytical methods confirm that hexagonal prism-shaped nanoparticles significantly increase cellular absorption in PC3 prostate cancer cells compared with spherical forms, according to a comparative investigation of cabazitaxel-loaded CSMS. Furthermore, drug-loaded CSMS increases intracellular ROS production and enhances cabazitaxel’s anticancer efficacy [144]. MSN-loaded with docetaxel (DTX-MSNs) has been studied for intertumoral therapy of localized prostate cancer. Intertumoral delivery of DTX-MSNs significantly decreased systemic drug exposure and related toxicity while exhibiting better antitumor activity than traditional intravenous docetaxel therapy in a human prostate cancer xenograft mice model. Compared with intratumorally administered free docetaxel, this nanomedicine improved therapeutic efficacy by increasing drug retention and accumulation within tumor tissues [145]. Chlorophyll-loaded MSN (Chl-MSNs) has been developed as a delivery system for photodynamic cancer treatment. The nanocarrier exhibited high chlorophyll encapsulation efficiency, physicochemical characteristics, improved photostability, and low dark toxicity. In vitro, it showed effective light-activated photodynamic activity under both red and blue laser irradiation in HepG2 liver cancer, MDA-MB-231 breast cancer, and human skin fibroblast cell lines [146]. A theranostic mesoporous silica nanoplatform, MCM-41-NH2-DTPA-Gd3^+^-MIH, was developed for simultaneous MRI. The MCM-41-based nanomaterial was functionalized with amine groups, conjugated with DTPA, complexed with gadolinium ions, and loaded with the mesoionic anticancer agent MIH 2.4Bl. In vitro studies showed selective cytotoxicity toward MDA-MB-231 breast cancer cells compared to normal mammary epithelial cells. In vivo MRI evaluation in a 4T1 breast tumor model confirmed effective tumor imaging [147]. Artemisinin-loaded PLGA electrospun nanofibers as a targeted drug delivery method for breast cancer treatment. Controlled degradation of these nanofibers and delayed drug release achieve long-lasting therapeutic benefits. By enhancing intracellular ROS formation and inducing apoptosis through regulation of Bax, caspase-3, and p53 expression, the nanofiber formulation exhibits greater cytotoxicity against SK-BR-3 breast cancer cells than free artemisinin [148]. Table 2 summarizes the physicochemical characteristics, experimental cancer models, mechanisms of action, and anticancer efficacy of representative MSN-based formulations. The compiled studies demonstrate that functionalized MSNs with optimized size, morphology, and surface modifications significantly enhance targeted drug delivery, promote apoptosis and ROS-mediated cytotoxicity, and improve therapeutic outcomes across diverse in vitro and in vivo cancer models. These findings highlight the strong potential of MSN-based nanocarriers as efficient, versatile platforms for advanced cancer therapy.

Despite significant advances in cancer nanomedicine, the therapeutic utility of targeted MSNs and the enhanced permeability and retention (EPR) effect remain debated. The EPR effect is consistently observed in rapidly growing murine tumors; however, because tumor vascularization, stromal composition, extracellular matrix density, interstitial fluid pressure, perfusion, and hemodynamics vary widely, its magnitude and reproducibility are much lower in human cancers [149]. Additionally, the universal efficacy of ligand-mediated active targeting is limited by the significant variation in the expression of molecular targets, such as integrins, folate receptors, transferrin receptors, and epidermal growth factor receptors, among patients, tumor types, and disease stages. Crucially, a significant percentage of actively targeted nanoparticles accumulate in the liver, spleen, and other MPS organs before reaching tumor tissues, and they typically maintain the biodistribution profile of their non-targeted counterparts [150]. Therefore, rather than significantly increasing overall tumor accumulation, active targeting primarily enhances cellular absorption following nanoparticle extravasation. To maximize their translational potential, future MSN-based cancer treatments should prioritize strategies that enhance tumor penetration, overcome stromal and vascular barriers, reduce off-target organ accumulation, and be validated in clinically relevant orthotopic and patient-derived tumor models.

**Table 2:** MSN-based anticancer formulations, physicochemical characteristics, experimental cancer models, mechanisms of action, and anticancer efficacy.

S. No.	MSN Formulation	Size (nm)	Shape	Therapeutic Drug	Cancer Model (Cell Line/In Vivo)	Mechanism of Action	Anticancer Efficacy	Ref.
1.	PEG-DOT-MSNs	100	Spherical core–shell	Doxorubicin	4T1 cells and female BALB/c mice model	ROS generation, apoptosis	Synergistic ferroptosis/chemotherapy/CDT therapy	[127]
2.	Folic acid-MSNs	100–200	Spherical	Doxorubicin	MDA-MB-231 cells	Apoptosis and primary necrosis	Chemotherapy and photodynamic therapy	[115]
3.	D-α-tocopherol-PEGylated MSNs	200	Spherical	Paclitaxel	B16F10 tumor-bearing mice	Microtubule disruption and apoptosis	Cytotoxicity and intracellular accumulation	[123]
4.	Chitosan-glycine-MSNs	330	Spherical	Colchicine	HCT116, HepG2, PC3 cells	Targeted drug delivery	Apoptosis and cytotoxicity	[118]
5.	Hyaluronic acid-modified MSNs	52	Spherical	Cisplatin	A549-Luc-C8 cancer cells	Cytotoxic, apoptotic, and oxidative Stress	Significant tumor inhibition and hemocompatibility	[110]
6.	HA-coated MSNs	155	Spherical	Doxorubicin	OS-bone cancer cells	Cellular uptake and ROS modulation	Intracellular release and cytotoxicity	[142]
7.	Gold-MSN hybrid	63–79	Core–shell	Epigallocatechin-3-gallate	NIR model	Photothermal therapy	Marked tumor regression	[151]
8.	Polydopamine-MSNs	87	Spherical	Umbelliprenin	MCF-7 breast cancer cells	Gene expression apoptotic pathways	Biocompatibility and cytotoxicity	[120]
9.	PEGylated MSNs	29–44	Spherical	Docetaxel	U87MG cells and BALB/c mice model	Caspase activation and apoptotic pathways	Drug delivery and antiproliferative effects	[141]
10.	Dihydroartemisinin-MSNs	100	Spherical	Tannic acid	HCT116 cells and animal model colon cancer	Cellular uptake and ROS apoptosis	pH-responsive release and cytotoxicity	[143]
11.	5-Fluorouracil-MSNs	907	Spherical	Folic acid	HeLa cervical cancer cells	pH-dependent release and cellular uptake	Enhanced growth inhibition	[137]
12.	Alginate-MSNs	120	Core–shell	Curcumin and quercetin	HT-29 colon cancer cells	pH- and redox-release and cytotoxicity	Synergistic anticancer activity	[119]
13.	Mercaptopropyltriethoxysilane-MSNs	71	Spherical	Tin	Balb/C female mice	ROS apoptosis and DNA damage	Significant tumor suppression	[131]
14.	Folic acid-CS-MSNs	62	Spherical	Piceatannol	MCF-7 breast cancer cells	Intracellular uptake and apoptosis	Cytotoxicity and hemocompatibility	[133]
15.	MSNs	159	Spherical	Docetaxel	LNCaP tumor-bearing mice	Cellular uptake and apoptosis	Reduce tumor volume and biocompatibility	[145]

### 4.3. Tissue Engineering and Regenerative Medicine

Britanin-loaded MSNs (britanin@MSNs) were developed. Mechanistically, autophagy and osteogenic protein production are enhanced by stimulation of the NPY1R-mediated PI3K/AKT/mTOR signaling pathway. Through the TGF-β1/SMAD3 pathway, the system lowers oxidative stress and increases M2 polarization in macrophages. It also increases angiogenic activity in endothelial cells by upregulating VEGF and CD31 expression. In a rat femoral defect model, britanin@MSNs dramatically enhance bone regeneration, mineral density, and tissue remodeling in vivo [152]. In a study, methacrylated copper-doped MSN (Cu@MSNMA) was incorporated into a GelMA/gelatin matrix to create a biomimetic nanocomposite hydrogel. Cu@MSNs were structurally modified to integrate stably into the hydrogel network, increasing porosity and crosslinking density. Higher nanoparticle content increased strength in mechanical tests, with a 5-weight-percent loading yielding the best results. Because of the bioactive effects of silicon and copper ions, both in vitro and in vivo studies have shown that the composite hydrogel significantly enhances antibacterial activity and promotes osteogenic differentiation [153]. Resveratrol-loaded MSNs (MSN@Res) can enhance resveratrol solubility, stability, and therapeutic efficacy in treating hypertrophic scars. By decreasing protective autophagy and triggering apoptosis in hypoxic environments, the MSN-based delivery method dramatically reduced hypertrophic scar fibroblast proliferation. Mechanistic studies show that MSN@Res reduces autophagy-related responses and increases programmed cell death by modulating the ROS-mediated p38-MAPK/HIF-1α/p53 signaling pathway [154]. Neodymium-doped MSN (NDMSN) has been developed as an immunomodulatory platform for bone regeneration. In both endothelial and mesenchymal stem cells, conditioned media from NDMSN-treated macrophages stimulated angiogenic and osteogenic responses. Additionally, in vivo studies showed that NDMSN enhanced bone regeneration, reduced inflammatory bone loss, and inhibited osteoclast activity [155].

To treat periodontitis, a triple-functional thermosensitive hydrogel (HQUP@TF127) containing quercetin-loaded hollow MSNs was developed. Characterization confirmed successful synthesis with well-defined structural and surface properties. The nanoparticles exhibited a uniform morphology, an appropriate particle-size distribution, and a stable surface charge. HQUP@TF127 successfully eliminated periodontal pathogens, decreased oxidative stress and inflammation, restored cellular homeostasis, and improved osteogenic differentiation in periodontal ligament stem cells. The hydrogel’s promise as an enhanced therapeutic platform for periodontal regeneration was highlighted by its in vivo suppression of osteoclast activity, facilitation of periodontal tissue regeneration, and enhancement of bone mineral density (Figure S2) [156]. Dendritic MSNs (DMSNs) have been synthesized and shown to stabilize Pickering emulsions in enhanced oil recovery (EOR) applications. Under difficult reservoir conditions, the high specific surface area and increased surface roughness of DMSNs encouraged robust interfacial adsorption and enhanced emulsion stability. Core flooding studies highlighted the promise of DMSN-stabilized Pickering emulsions for cutting-edge EOR technologies, showing a significant increase in oil recovery efficiency (27.6%) [157]. A polydopamine-coated, diselenide-bridged MSN platform (PDA-DSeMSN@CAPE) was developed to treat secondary brain injury after intracerebral hemorrhage. This multifunctional nanocarrier enables controlled release of caffeic acid phenethyl ester under oxidative stress by combining ROS-responsive drug delivery with intrinsic ROS-scavenging capabilities. PDA-DSeMSN@CAPE dramatically decreased oxidative stress, polarized microglia from the pro-inflammatory M1 phenotype to the anti-inflammatory M2 phenotype, and inhibited neuroinflammation [158]. To improve antioxidant and anti-inflammatory efficacy, quercetin and cerium oxide nanozymes were combined to create CeO_2_/Que)@mSiO_2_. The large-pore mesoporous silica framework enabled high quercetin loading and prolonged drug release, addressing the poor solubility and bioavailability of free quercetin. Biological tests showed effective scavenging of reactive oxygen species and notable inhibition of pro-inflammatory cytokines [159].

**Figure 6:**
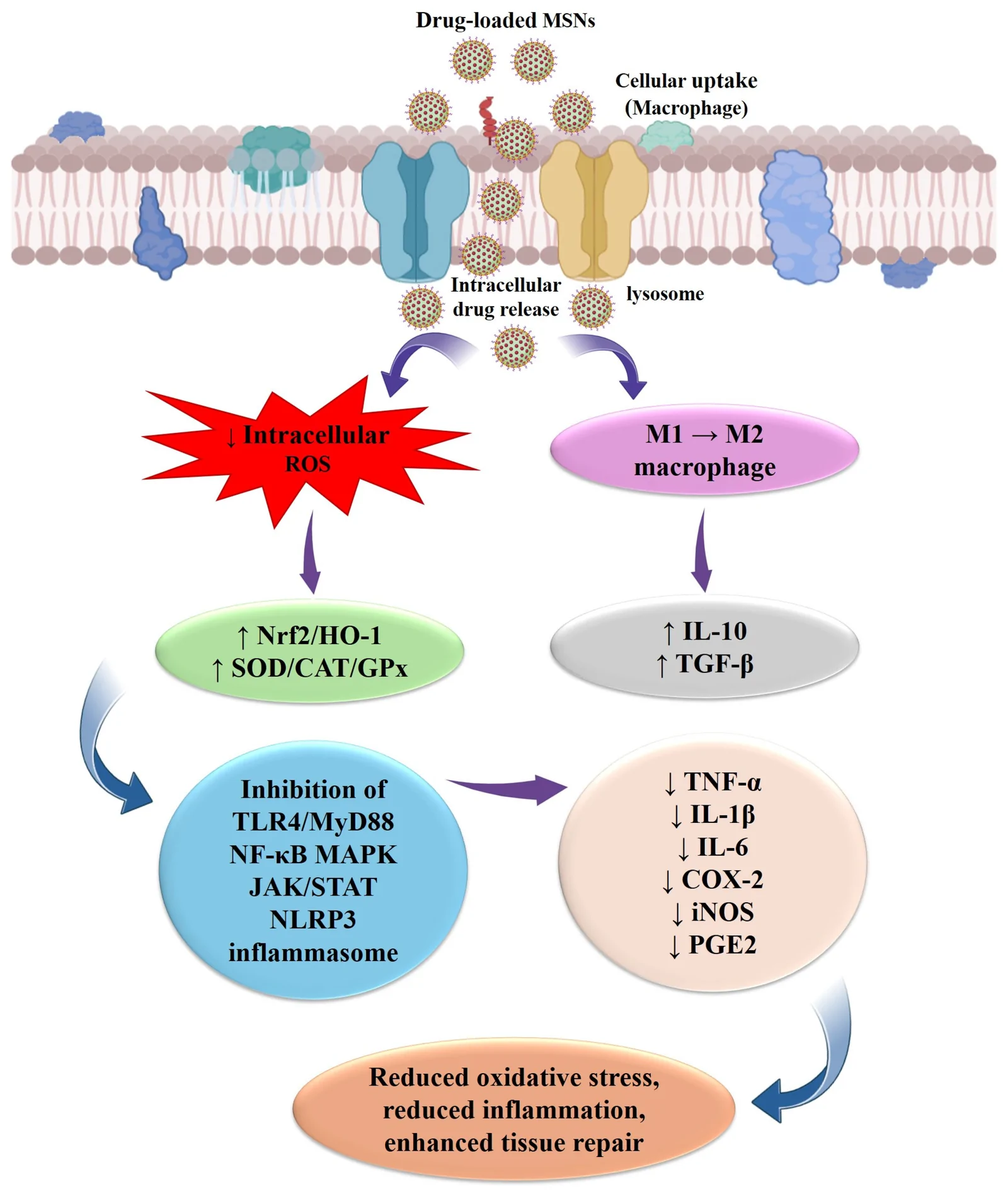
Schematic illustration of the anti-inflammatory mechanism of drug-loaded MSNs. Following cellular uptake and intracellular drug release, MSNs reduce oxidative stress, promote macrophage polarization from M1 to M2, activate antioxidant defenses (Nrf2/HO-1), and suppress the NF-κB/MAPK/NLRP3 inflammatory signaling pathways, thereby decreasing pro-inflammatory cytokine production. Collectively, these effects establish an anti-inflammatory microenvironment that enhances angiogenesis, re-epithelialization, and tissue regeneration, thereby accelerating tissue repair and wound healing (created using BioRender.com).

A study developed an injectable, self-healing manganese-doped MSNs-loaded dual-crosslinked hydrogel (Mn-MSN@Gel) for rotator cuff tendon-bone interface regeneration. The hydrogel demonstrated favorable mechanical properties, biocompatibility, biodegradability, and sustained Si^4+^ and Mn^4+^ release. Mn-MSN@Gel enhanced stem cell migration, proliferation, osteogenic and tenogenic differentiation, while reducing oxidative stress and inflammation. In vivo, it improved biomechanical properties and promoted tendon-bone tissue regeneration, demonstrating potential for rotator cuff repair [160]. A study developed naringenin-loaded manganese-doped MSNs (MMSN@NAR) to regulate macrophage polarization for atherosclerosis treatment. MMSN@NAR demonstrated high drug-loading capacity, pH-responsive release, and good biocompatibility. In vitro, it preferentially accumulated in M1 macrophages, reduced inflammatory and oxidative stress responses, and promoted M2 polarization through the AMPK pathway. In mice, MMSN@NAR accumulated in atherosclerotic plaques and reduced plaque area, highlighting its therapeutic potential [161]. Reduced oxidative stress and inflammation, in turn, accelerate tissue regeneration and functional wound healing by promoting angiogenesis, fibroblast proliferation, collagen synthesis, extracellular matrix remodeling, and re-epithelialization. These complex processes highlight the significant therapeutic potential of drug-loaded MSNs as cutting-edge anti-inflammatory nanoplatforms for managing inflammatory conditions and for regenerative medicine applications (Figure 6).

MSNs loaded with nanoceria (Ce@MSNs) as a multifunctional therapeutic platform for treating osteoporosis. Ce@MSNs showed strong ROS-scavenging activity, significantly reducing oxidative stress while preserving biocompatibility and effective cellular uptake. Ce@MSNs increased osteogenic responses in osteoblast–macrophage co-culture systems and stimulated osteoblast proliferation and mineralization in the absence of osteogenic supplements. These nanoparticles also altered oxidative-stress pathways involved in bone remodeling [162], leading to the development of arginine-loaded MSNs (Arg@MSNs) as functional additives for orthodontic adhesives. Arg@MSNs were included to maintain polymerization and bonding performance while enabling prolonged arginine release. The Arg@MSN-containing glue maintained therapeutically relevant adhesive strength and showed biocompatibility. These findings support the potential of MSN-based dental biomaterials as multipurpose platforms for the regulated distribution of bioactive agents and the prevention of enamel demineralization during orthodontic treatment [163]. To treat implant-related infections, an MSN system (MRL) loaded with LL-37 and modified by RGD was created. This nanoplatform combines immunomodulatory properties with the direct antibacterial activity of the peptide LL-37 to treat biofilm-associated bacterial infections. While RGD surface modification mitigates excessive macrophage pyroptosis and maintains overall immune function, LL-37 degrades bacterial membranes and promotes macrophage polarization toward the antimicrobial M1 phenotype. Studies show that MRL improves infection control, reduces biofilm-induced immunosuppression, and increases bacterial clearance [164].

**Figure 7:**
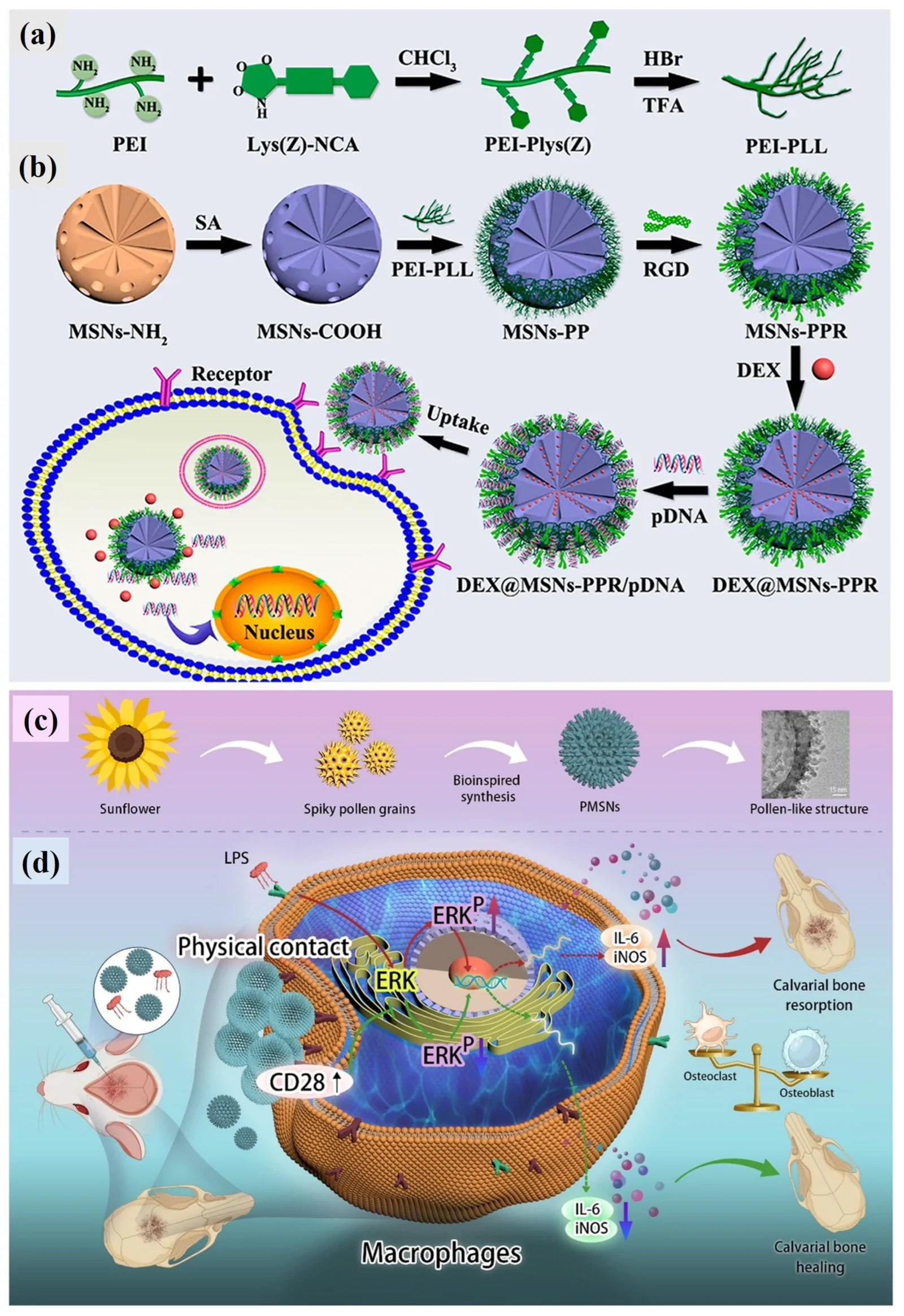
(**a**) Synthetic route for the preparation of PEI–PLL copolymers. (**b**) Schematic illustration of the fabrication of PEI–PLL- and RGD-functionalized MSNs for the co-delivery of plasmid DNA (pDNA) and therapeutic agents into target cells. Reproduced with permission from [165], under the CCC License, American Chemical Society. (**c**) The bioinspired fabrication of PMSNs and their unique surface architecture is designed to enhance biological interactions, and (**d**) the PMSNs modulate macrophage-mediated inflammation via CD28 signaling, promoting an anti-inflammatory environment that supports osteogenic differentiation and efficient bone regeneration. Reproduced from [166] under the CC BY 4.0 License, Elsevier.

RGD-functionalized MSNs coated with polylysine-modified polyethylenimine (PEI–PLL) have been developed for the co-delivery of bone morphogenetic protein-2 (BMP-2) plasmid DNA and dexamethasone (DEX). The resulting nanocarrier exhibited high cytocompatibility, effective gene transfection, rapid plasmid DNA release, and prolonged dexamethasone release. Bone mesenchymal stem cells (BMSCs) successfully expressed BMP-2 in vitro, resulting in improved osteogenic differentiation, as evidenced by increased calcium deposition, upregulated osteogenic gene expression, and higher alkaline phosphatase activity (Figure 7a,b) [165]. Pollen-like MSN (PMSNs) have been shown to influence macrophage immunological responses and promote bone repair through surface topology-dependent processes. By increasing CD28 expression and reducing ERK phosphorylation, PMSNs prevent M1 macrophage polarization and reduce pro-inflammatory marker production, in contrast to traditional smooth MSN. PMSNs’ unique surface shape alters interactions between nanoparticles and cell membranes, increasing anti-inflammatory responses without requiring nanoparticle phagocytosis. Furthermore, as demonstrated by elevated mineralization and BMP2 expression, conditioned media from PMSN-treated macrophages considerably stimulate osteogenic differentiation of bone marrow-derived stromal cells (Figure 7c,d). PMSN-treated anti-inflammatory macrophages greatly enhanced mBMSC osteogenic differentiation. After treatment, BMP-2 expression increased, along with mineralized matrix deposition and osteogenic markers [166].

Calcium and gallium-doped MSNs were developed and evaluated for their potential in bone-related applications. Under physiological conditions, gallium showed sustained release, while calcium incorporation altered the nanoparticle morphology and surface characteristics. In vitro, gallium-containing MSNs promoted pre-osteoblast proliferation and osteogenic differentiation, while also reducing osteoclast differentiation. These findings suggest potential osteogenic and anti-osteoclastic effects of the nanoparticles, although further in vivo studies are required to confirm their role in bone regeneration and resorption [167]. Fibronectin-coated, thioketal-modified MSNs loaded with epigallocatechin-3-gallate (EGCG) demonstrated ROS-responsive drug release and excellent biocompatibility for diabetic nephropathy therapy. The multifunctional nanoplatform effectively reduced oxidative stress, suppressed apoptosis and M1 macrophage polarization, enhanced autophagy, and significantly improved renal function and tissue repair in both in vitro and streptozotocin-induced diabetic mouse models [168]. This emulsion combines *Mesona chinensis* essential oil and betanin-loaded quaternary ammonium-functionalized MSNs, which provide potent antioxidant activity. Adding the emulsion improved the films’ physicochemical features, including reduced water content and improved solubility, altered surface morphology, and enhanced barrier properties. Superior DPPH and ABTS radical-scavenging activity indicated that the resulting composite films had far higher free-radical scavenging activity than the control films [169].

Etoricoxib-loaded MSN (ETB-MSNPs) have been developed and incorporated into a carbopol–chitosan gel. Etoricoxib was effectively encapsulated within the mesoporous silica nanocarrier, resulting in enhanced skin penetration, prolonged drug release, and improved solubility. Significant decreases in pro-inflammatory cytokines, including TNF-α and IL-1β, indicated that the tailored ETB-MSNPs gel exhibited greater anti-inflammatory activity than the traditional etoricoxib gel [170]. Aptinin-loaded MSNs have been developed as enhanced hemostatic agents for rapid bleeding control. The modified nanoparticles’ high drug encapsulation efficiency and prolonged aprotinin release enhanced therapeutic performance. Studies showed a considerable reduction in blood loss, hemostasis time, and blood clotting time compared with traditional treatments. Histopathological examinations also showed the formulation’s non-toxicity and biocompatibility [171]. Aminated three-dimensional dendritic MSNs as a sophisticated nanocarrier system for delivering simvastatin. These nanoparticles have a high drug-loading capacity, consistent nanoscale size, and a positive surface charge. An in vivo study using a hyperlipidemia model demonstrated significant antioxidant activity, improved antihyperlipidemic efficacy, and protection against oxidative tissue damage. Histopathological examinations confirmed positive biocompatibility and tissue safety [172]. Piperine-loaded MSNs (PIP-MSNs) have been developed as a potential protoscolicidal agent for the treatment of cystic echinococcosis. At the evaluated concentrations, the nanocarrier exhibited good physicochemical properties, effective drug encapsulation, and prolonged piperine release, while retaining biocompatibility and cell survival above 90%. In vitro, PIP-MSNs showed significant, dose-dependent protoscolicidal action against Echinococcus granulosus protoscoleces, achieving up to 94.67% mortality [173].

A study showed that magnesium-modified MSNs exhibited improved biodegradability and regulated release of bioactive ions for regenerative medicine. Adding Mg^2+^ ions makes the siloxane network less stable, allowing controlled breakdown while preserving the mesoporous structure. Magnesium and silicon ion release improves mitochondrial function, reduces oxidative stress, boosts cellular antioxidant capacity, and controls macrophage inflammatory responses. In vivo experiments demonstrate improved tissue regeneration in periodontal defects [174], highlighting the potential of biodegradable Mg-doped MSNs as multipurpose biomaterials for regenerative therapies. The development of a mineralized chitosan–alginate–gelatin (CAG) biocomposite scaffold for bone tissue engineering that incorporates dexamethasone-loaded MSNs has been described. This scaffold combines prolonged dexamethasone release with calcium phosphate surface mineralization, resulting in increased mechanical strength, less swelling, and improved structural stability. Mesenchymal stem cells generated from bone marrow showed enhanced proliferation and osteogenic differentiation in vitro. Compared with non-mineralized and non-drug-loaded controls, in vivo evaluations revealed significantly greater new bone formation [175]. Figure S3 presents key strategies to enhance the biomedical performance and safety of MSNs. Surface functionalization with polymers, proteins, and liposomes enhances biocompatibility and reduces toxicity. Biomimetic MSNs coated with cell membranes enable active targeting of diseased areas, prolong circulation, and aid immune evasion. Furthermore, stimulus-responsive gatekeepers enable controlled medication release in response to internal or external stimuli, including pH, redox conditions, temperature, light, ultrasound, and enzymes. Major pathways of MSN-induced toxicity include oxidative stress, autophagy dysregulation, lysosomal degradation, inflammasome activation, and pro-inflammatory cytokine release. This study highlights the need for logical surface engineering to ensure safe clinical translation [176].

## 5. MSNs in Wound Healing Applications

MSNs are promising multifunctional platforms for wound healing owing to their distinctive physicochemical properties, high drug-loading capacity, controlled-release profiles, and adaptable surface functionalization. By enabling the targeted release of growth factors, anti-inflammatory substances, antibacterial agents, and other bioactive compounds directly at wound sites, MSN-based delivery systems enhance therapeutic efficacy and reduce systemic side effects. MSNs’ ability to release sustained, stimuli-responsive substances supports long-term antibacterial activity and efficient destruction of microbial biofilms, which are major barriers to chronic wound healing [177]. Furthermore, MSNs can accelerate tissue repair and regeneration by modulating the inflammatory milieu, encouraging macrophage polarization toward regenerative phenotypes, stimulating angiogenesis, and improving cellular proliferation and extracellular matrix remodeling. Integrating MSNs into cutting-edge wound care platforms, including hydrogels, nanofibrous scaffolds, composite dressings, and microneedle systems, has facilitated the development of multifunctional wound therapies with improved moisture retention, mechanical stability, bioactivity, and localized therapeutic delivery [178]. Overall, these characteristics make MSNs highly promising nanomaterials for treating both acute and chronic wounds and for developing cutting-edge wound-healing techniques. An intelligent MSN was developed as both a structural and functional mediator between a pH-sensitive hyaluronic acid–gentamicin hydrogel. The dressing enables non-invasive, real-time monitoring of the wound-healing process through visible color changes caused by local pH differences, shifting from light yellow to green and back to bright yellow as healing progresses. By combining antibacterial therapy with visual, on-site assessment of healing, this multipurpose MSN-based hydrogel offers a promising foundation for intelligent wound management [179]. L-arginine-modified sodium alginate and guar gum matrix was combined with thiol-functionalized MSN. While the polysaccharide mixture maintains structural integrity and fosters a favorable microenvironment for wound healing, L-arginine functionalization promotes collagen production and epithelialization. Physicochemical and biological analyses indicate that the material has qualities appropriate for wound applications. In experimental models, the film shows high compatibility and can facilitate rapid wound closure [180].

A multifunctional Ag@MSN-based nanoplatform (AMPC@siTNF-α), co-loaded with ciprofloxacin (CFL) and TNF-α small interfering RNA (siTNF-α), was developed for the treatment of bacterial wound infections. While siTNF-α successfully inhibited TNF-α expression in macrophages, thereby reducing inflammation and promoting tissue healing, the released Ag+ ions and CFL showed potent antibacterial activity against Escherichia coli. AMPC@siTNF-α significantly accelerated wound healing and prevented suppuration in a mouse model of infected wounds. The nanoplatform demonstrated low toxicity in vitro and in vivo, suggesting its potential as a synergistic therapeutic approach for treating infected wounds (Figure S4a,b). In vivo studies demonstrated that the optimized formulation significantly accelerated the healing of *E. coli*-infected wounds, as shown by enhanced wound closure and improved tissue regeneration [181]. The development of liquid crystal-modified MSNs (LC–MSNs) incorporated into an injectable hydrogel dressing designed to enhance wound healing and tissue regeneration has been described. Human umbilical vein endothelial cells (HUVECs) proliferate, migrate, and exhibit angiogenic activity when the bioactivity of MSNs is significantly enhanced by LC modification. Incorporating LC–MSNs into a hydrogel matrix and applying them to full-thickness wounds expedited tissue repair by promoting granulation tissue development, collagen deposition, and neovascularization (Figure S4c). In a full-thickness wound model, LC–MSN hydrogel treatment significantly accelerated wound closure and enhanced tissue regeneration [182].

Colloidal-MSNs were incorporated into a gelatin–tannic acid (GTA) matrix cross-linked with silver ions (GTA/Ag) to create a multifunctional bioadhesive hydrogel. Through polyphenol–metal coordination, this system generates strong wet adhesion, and CMSNs promote the physical adsorption of polymer chains and tissue biomolecules, further strengthening tissue bonding. Elastic modulus and moist tissue adhesion strength on pig skin significantly increased following CMSN inclusion. Additionally, the composite showed cytocompatibility with fibroblasts, biodegradability, and antioxidant activity [183]. Vancomycin-loaded MCM-41 MSNs and malic acid-coated magnetite nanoparticles were combined to create an alginate-based hydrogel wound dressing. The resulting hybrid film exhibited controlled vancomycin release, suitable swelling behavior, and structural homogeneity. The dressing exhibited increased antibacterial activity attributable to the combined actions of the mesoporous silica carrier and magnetite nanoparticles. Magnetite incorporation enabled magneto-responsive smart wound dressings, allowing external magnetic stimulation [184]. Dragon’s Blood (DB)-loaded mesoporous silica nanoparticles (DB-MSNs) were developed as a multifunctional hemostatic and antibacterial nanoplatform to improve DB solubility and enable rapid release. DB-MSNs enhanced blood coagulation and exhibited greater hemostatic activity than DB or MSNs alone, while their antibacterial activity against *S. aureus* and *E. coli* increased with increasing DB content. Among the tested DB-MSN formulations, 10DB-MSN exhibited the strongest hemostatic effect, together with good hemocompatibility and cytocompatibility, indicating its potential for rapid hemostatic and antibacterial applications in wound and trauma management [185].

**Figure 8:**
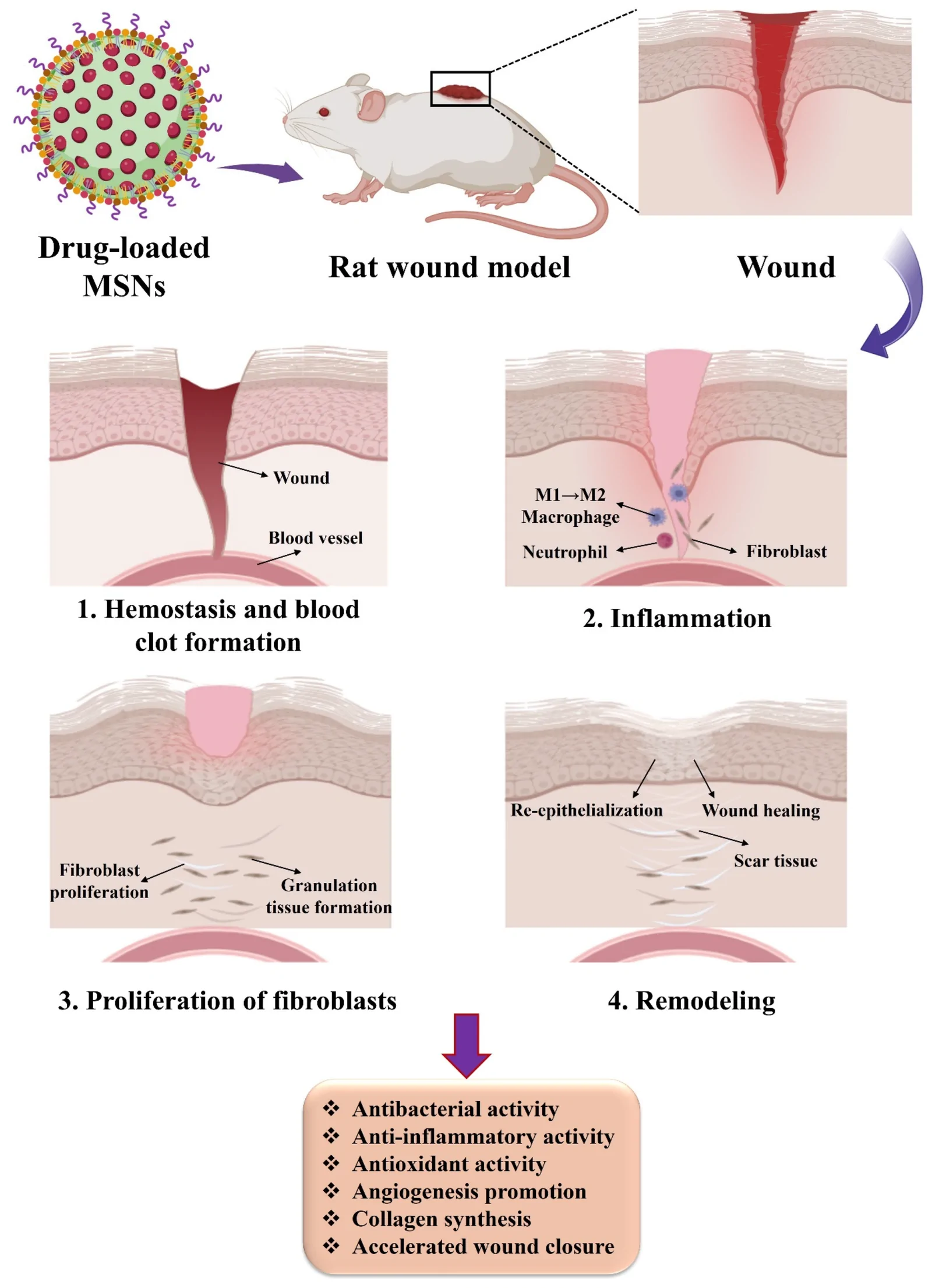
Schematic illustration of the therapeutic role of drug-loaded MSNs in a rat wound model during the sequential phases of wound healing, including (**1**) hemostasis and blood clot formation, (**2**) inflammation, (**3**) fibroblast proliferation, and (**4**) tissue remodeling. Drug-loaded MSNs accelerate wound repair by exerting antibacterial, anti-inflammatory, and antioxidant effects and promoting wound closure. These coordinated biological responses ultimately enhance tissue regeneration and minimize scar formation (created using BioRender.com).

Drug-loaded MSNs represent promising therapeutic nanoplatforms for wound healing by modulating key biological processes throughout the sequential phases of tissue repair (Figure 8). Drug-loaded MSNs provide controlled, prolonged release of therapeutic substances at the wound site after local injection, thereby accelerating hemostasis, promoting blood clot formation, and reducing microbiological contamination [186]. By eliminating pathogenic microorganisms, scavenging excess ROS, and modulating inflammatory responses by polarizing macrophages from the pro-inflammatory M1 phenotype to the pro-regenerative M2 phenotype, MSNs demonstrate antibacterial, anti-inflammatory, and antioxidant properties during the inflammatory phase. By suppressing persistent inflammation, this immunomodulatory activity fosters a milieu that is conducive to tissue regeneration [187]. MSNs accelerate tissue regeneration by promoting fibroblast proliferation, angiogenesis, collagen deposition, extracellular matrix remodeling, and granulation tissue formation during the proliferative phase. Improved collagen maturation and re-epithelialization aid in wound contraction, restoration of tissue architecture, and reduction in excessive scar formation during the remodeling phase [188]. The multifunctional therapeutic qualities of drug-loaded MSNs—such as immunomodulation, controlled drug release, antimicrobial activity, antioxidant effects, and promotion of tissue regeneration—significantly accelerate wound closure and demonstrate their enormous potential as cutting-edge nanotherapeutic platforms for treating acute, chronic, and infected wounds.

A multifunctional ionic composite hydrogel was created that included L-arginine, glucose oxidase-loaded hollow MSNs (HMSNs-GOx), and an ionic liquid based on FeCl_3_/DDBAC. While L-arginine-mediated nitric oxide synthesis promoted angiogenesis, the HMSNs-GOx component enabled sustained glucose control by shielding glucose oxidase from inactivation in the diabetic wound milieu. The hydrogel showed considerable promise as a multi-target healing therapy platform for treating diabetic wounds by modulating pro-regenerative signaling pathways [189]. The development of 3D-printable nanocomposite hydrogels incorporating surface-modified MSNs to deliver therapeutic proteins has been described. Results show that MSN surface charge plays a crucial role in controlling biological activity, immobilization stability, and protein adsorption. Enzyme stability and activity retention were higher with near-neutral and negatively charged MSNs than with positively charged MSNs. Furthermore, the study used 3D printing to create enzyme-loaded nanocomposite hydrogels, highlighting their promise as adaptable biomolecule delivery systems for tissue engineering and wound healing [190]. A multifunctional microneedle (MN) patch (PFG/M MNs) was developed to treat infected wounds. The MN tips were loaded with Fe/PDA@GOx@HA nanoparticles containing glucose oxidase. Amine-modified MSN (AP-MSNs) were integrated into the MN bases to offer anti-inflammatory properties. By synergistically modulating bacterial burden and the wound immune microenvironment, the PFG/M MN system significantly accelerated infected wound healing. It demonstrated substantial potential as an advanced therapeutic strategy for clinical wound management [191].

A study developed an electrospun composite nanoscaffold composed of polycaprolactone, amine-functionalized SBA-15 MSN, and curcumin. Curcumin added antibacterial qualities, and amine-functionalized SBA-15 enhanced biocompatibility and cellular adhesion. In vivo studies in Wistar rats showed nearly complete wound closure within 21 days, along with increased re-epithelialization, collagen deposition, and granulation tissue formation, supporting its potential as an advanced wound-healing biomaterial [192]. A GelMA/SFMA composite hydrogel containing platelet-derived extracellular vesicles (PDEVs) and resveratrol-loaded MSNs has been developed. This hydrogel showed good mechanical strength, minimal cytotoxicity, long-term drug release, and biodegradability. By lowering iNOS expression and promoting angiogenesis, the formulation inhibited macrophage-mediated inflammatory responses in vitro, as demonstrated by better tube formation in human umbilical vein endothelial cells. The hydrogel boosted anti-inflammatory markers (TGF-β1 and Arg-1), decreased pro-inflammatory mediators (TNF-α and iNOS), accelerated wound closure, and encouraged neovascularization in a diabetic wound model [193]. A study has developed CMCS/gelatin composite films incorporating myrtle extract-loaded MSNs for wound-healing applications. In addition to improving the dressings’ mechanical properties, swelling behavior, and moisture retention, adding MSNs enables continuous release of the bioactive extract. These films exhibit lower cytotoxicity toward fibroblast cells and biocompatibility. In vivo studies demonstrate enhanced wound contraction, fibroblast migration, and collagen deposition, all of which speed up wound healing [194]. The development of wound dressings based on bacterial nanocellulose combined with MSNs for continuous monitoring of wound pH has been described. The MSNs enable rapid colorimetric detection of wound pH changes associated with infection. This smart dressing platform enables real-time monitoring of wound status and offers opportunities to integrate therapeutic drugs in the future to improve chronic wound management [195].

**Figure 9:**
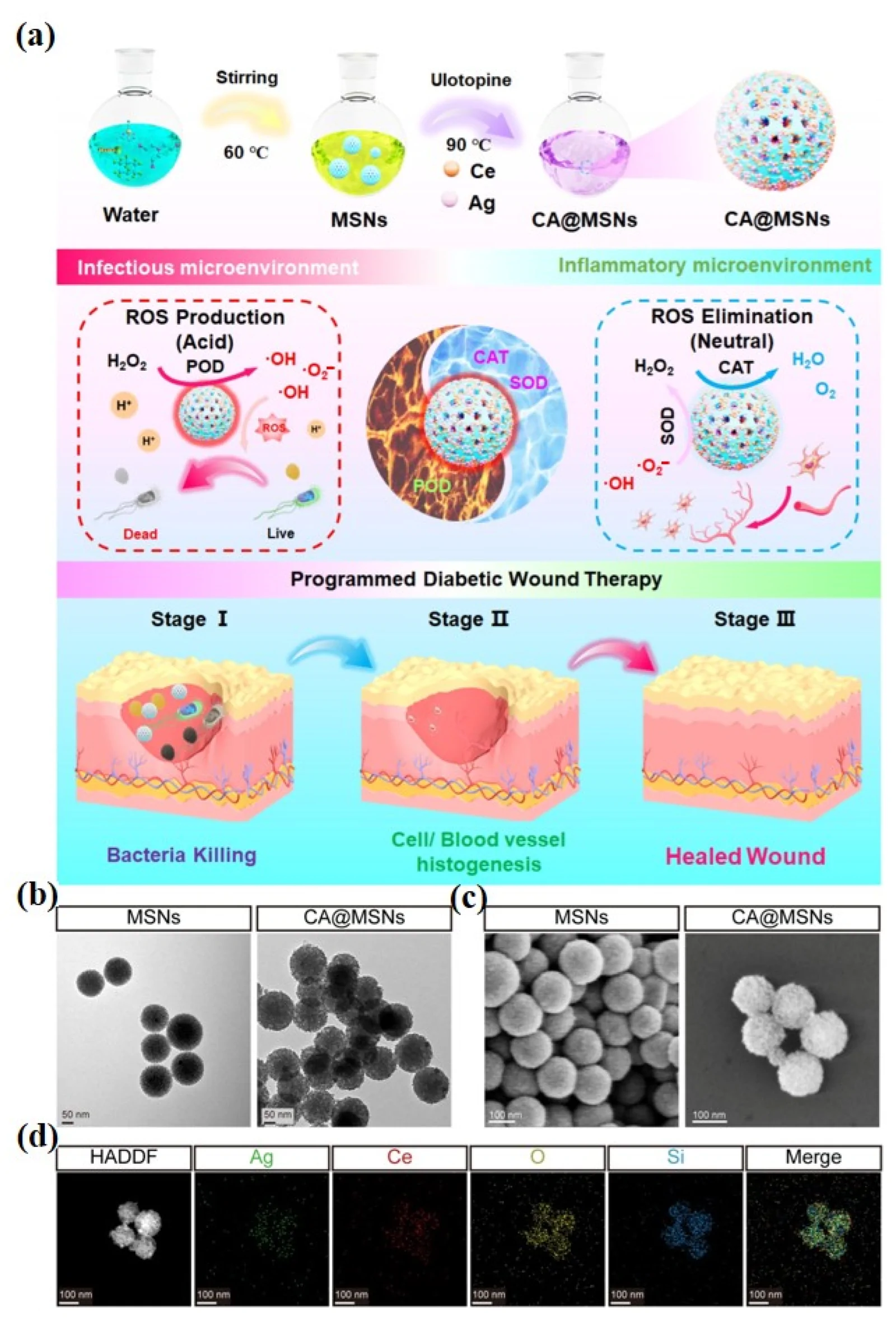
(**a**) Schematic illustration of the synthesis of CA@MSNs and their microenvironment-responsive ROS-generating and scavenging activities for the treatment of MRSA-infected diabetic wounds. (**b**) TEM and (**c**) SEM images showing the morphology of CA@MSNs. (**d**) HAADF-STEM image and corresponding EDS elemental mapping of CA@MSNs. Reproduced from [198], licensed under CC BY 4.0, MDPI.

A rutin-loaded MSN gel mixed with zinc oxide (Z-R/MSN) was used for burn wound healing. When the Z-R/MSN gel was applied topically, the wound contracted almost completely, significantly surpassing the control treatment. Its promise as an advanced topical dressing for burn wound management was highlighted by enhanced therapeutic efficacy, attributed to the combined anti-inflammatory and wound-healing properties of rutin, zinc oxide, and the MSN-based delivery system [196]. Chitosan-based skin patches containing doxycycline-loaded MSNs were used for wound-healing applications. In a rat wound model, this MSN-mediated delivery method enhanced collagen fiber organization, promoted neovascularization, reduced inflammation, and accelerated re-epithelialization. In the early phases of recovery, wounds treated with the patches closed considerably faster than those in the control group. Doxycycline-loaded MSN/chitosan patches may be an efficient means of enhancing skin wound healing, as the combined effects of chitosan and doxycycline improve tissue regeneration and wound repair [197]. Ce/Ag-doped MSNs (CA@MSNs) for the treatment of MRSA-infected diabetic wounds have been reported. By trapping bacteria and generating reactive oxygen species that mimic peroxidase activity, this tailored nanoplatform exhibits strong antibacterial activity and eradicates harmful bacteria. CA@MSNs reduce oxidative stress and promote endothelial cell migration by eliminating excessive reactive oxygen species in inflammatory microenvironments. CA@MSNs therapy dramatically lowers inflammation, increases collagen deposition and fibroblast activation, and accelerates wound healing in diabetic mouse models (Figure 9) [198].

A study developed electrospun chitosan nanofibrous scaffolds reinforced with MSNs and adorned with AgNPs. Scaffolds containing 7% AgNPs outperformed other formulations in mechanical strength and therapeutic efficacy. In vivo experiments showed increased fibroblast proliferation, collagen deposition, and neovascularization, which hastened wound healing. These findings suggest that silver-functionalized mesoporous silica/chitosan nanofibers have great promise as wound dressings for treating diabetic wounds [199]. Another study has fabricated hybrid membranes based on carboxymethyl cellulose (CMC) that include hyperbranched polysiloxane-functionalized MCM-41@HBPSi loaded with clindamycin hydrochloride. These hybrid membranes exhibited long-lasting antibacterial activity and biocompatibility. In vivo studies highlighted the potential of drug-loaded mesoporous silica/CMC composite membranes as cutting-edge antibacterial wound dressings, demonstrating faster wound closure with these materials [200]. A gel filled with MSN and containing Rosmarinus officinalis extract was used for wound-healing applications. The improved formulation’s excellent spreadability and sustained drug release enabled long-term delivery of the bioactive extract. This gel is a promising approach for enhancing wound healing and wound care management because it combines the therapeutic properties of rosemary extract with the controlled-release properties of mesoporous silica [201]. A bifunctional MSN was covalently conjugated to the anti-inflammatory medication ibuprofen and to either levofloxacin or norfloxacin. Enzymatic cleavage of amide bonds enabled controlled in situ release of both therapeutic substances. By delivering drugs locally and sustainably, the dual-functional nanocomposite fabrics show promise as cutting-edge medical bandages that can prevent infection and encourage wound healing [202]. MSNs encapsulating gold nanorods (GNRs@QMSN) were used for the treatment of bacterially infected diabetic lesions. This nanoplatform achieves excellent photothermal conversion efficiency by combining effective photothermal therapy mediated by gold nanorods. GNRs@QMSN showed promise as an advanced multifunctional treatment system for chronic wound infections, significantly accelerating wound healing by controlling infection in a diabetic wound infection model [203].

The development of a multifunctional hydrogel based on oxidized hyaluronic acid that combines metformin- and tetracycline-loaded MSNs for the treatment of diabetic wounds. This hydrogel showed strong antibacterial activity, high adhesiveness, and controlled dual-drug release, delivering rapid anti-inflammatory benefits and long-lasting antibacterial protection. Diabetic mice in vivo showed faster wound healing, with a 96.6% wound closure rate [204]. To treat chronic wounds, the study used a multifunctional fractal MSN-based nanoplatform to co-deliver honokiol and baicalin. In infected wound settings, folic acid-functionalized, chitosan-capped nanoparticles enabled drug release and targeted adherence to bacterial biofilms. Combining antibacterial and anti-inflammatory properties, the dual-drug nanoplatform greatly improved wound healing and showed great promise as a cutting-edge therapeutic approach for managing chronic wounds [205]. PLNC8 αβ, a protease-resistant antimicrobial peptide, was investigated for the treatment of infected wounds using functionalized nanocellulose dressings. The peptide was either directly adsorbed onto bacterial cellulose and TEMPO-oxidized nanocellulose or encapsulated within MSNs incorporated into the dressings. MSN-functionalized dressings exhibited enhanced peptide-loading capacity, sustained release, improved antimicrobial activity, and low cytotoxicity toward human fibroblasts and keratinocytes. In a porcine wound infection model, PLNC8 αβ demonstrated strong antimicrobial activity, effectively eradicating the infection and promoting wound re-epithelialization [206]. A multifunctional nanofibrous matrix comprising polygalacturonic acid, polyvinyl alcohol, capsaicin, and zinc-doped mesoporous silica (Zn/MCM-41) has been developed. This composite nanofiber shows enhanced cellular adhesion and proliferation, controlled drug release, and favorable mechanical properties. Zn/MCM-41 and capsaicin exhibit synergistic anti-inflammatory and regenerative effects that promote tissue repair and hasten wound healing [207]. Table 3 summarizes recent advances in MSN-based wound-healing systems designed to enhance tissue regeneration through the controlled delivery of antibiotics, phytochemicals, metal ions, and other bioactive molecules. The reported formulations consistently accelerated wound closure and enhanced collagen deposition, angiogenesis, re-epithelialization, and infection control, particularly in chronic and diabetic wound models. Furthermore, surface functionalization and multifunctional hydrogel or nanofiber-based MSN platforms significantly improved therapeutic efficacy by providing sustained drug release, antibacterial activity, and modulation of the inflammatory microenvironment. Collectively, these findings highlight the considerable potential of engineered MSN-based wound dressings as next-generation biomaterials for advanced wound management and future clinical translation.

## 6. Application of MSNs in Disease Diagnosis

MSNs have emerged as adaptable nanoplatforms for disease diagnosis and theragnostic applications due to their tunable pore structure, biocompatibility, and capacity for diverse surface modifications. These features enable the effective integration of imaging probes, contrast agents, targeting ligands, and therapeutic agents within a single platform. MSN-based imaging systems have been extensively researched for magnetic resonance imaging (MRI), fluorescence imaging, computed tomography (CT), positron emission tomography (PET), photoacoustic imaging, ultrasound imaging, and multimodal imaging. By enabling selective recognition of disease biomarkers, functionalizing MSN surfaces with antibodies, peptides, aptamers, or small-molecule ligands improves target specificity. It increases imaging sensitivity, spatial resolution, and early disease detection. Consequently, MSN-based theragnostic systems show great promise for neurodegenerative, infectious, and inflammatory diseases, as well as precision oncology [7].

Recent studies indicate that MSN-based imaging platforms substantially enhance disease visualization, molecular diagnosis, and therapeutic monitoring. For ovarian cancer biomarkers such as CA125 and HE4, biotin-functionalized dendritic MSNs integrated into multiplex lateral flow immunoassays have demonstrated high sensitivity and strong agreement with traditional clinical procedures [208]. Additionally, by boosting contrast, stabilizing signals, and encouraging target-specific accumulation, Gd-, Mn-, Fe_3_O_4_-, and fluorescent dye-functionalized MSNs have enhanced MRI and fluorescence imaging. Protein corona formation, nonspecific tissue accumulation, limited tissue penetration, potential long-term toxicity of imaging agents, and inadequate pharmacokinetic and biodistribution data remain major challenges to clinical translation [209]. Future research should prioritize developing biodegradable, multimodal, clinically translatable MSN-based theragnostic systems with improved targeting, long-term biosafety, standardized imaging performance, and validation in extensive clinical trials.

MSN-based platforms have shown great promise for diagnosing infectious diseases and other pathological disorders in addition to cancer. With improved accuracy and faster response times, functionalized MSNs can serve as highly sensitive biosensing systems for detecting proteins, nucleic acids, and disease-specific biomarkers [210]. MSN-assisted signal amplification significantly boosts analytical sensitivity when compared to traditional diagnostic techniques in MSN-based fluorescence diagnostic systems for viral pathogen identification. Furthermore, incorporating metallic and magnetic elements into MSN-based nanostructures has enabled multifunctional diagnostic agents with improved imaging contrast, stability, and bioinert properties. Taken together, these developments highlight the growing importance of MSNs as next-generation diagnostic nanoplatforms, offering enhanced sensitivity, multiplex detection, and multimodal imaging capabilities for early disease monitoring and diagnosis [211].

## 7. Biocompatibility, Toxicity, and Safety of MSNs for Biomedical Applications

The biocompatibility and biosafety of MSNs are critical for their clinical translation. Because of their tunable pore architecture, controllable surface chemistry, and biodegradable silica framework, MSNs are promising inorganic nanocarriers for biomedical applications. Factors such as particle size, morphology, pore structure, silica condensation, surface charge, and functionalization influence MSN biological behavior [212]. These characteristics collectively affect protein corona formation, cellular uptake, biodistribution, biodegradation, and clearance. While porous silica frameworks gradually degrade into orthosilicic acid under physiological conditions, the degradation rate depends on particle composition and structural features. Larger MSNs (>100 nm) or those with highly condensed silica frameworks typically biodegrade more slowly, show reduced renal clearance, and accumulate preferentially in the mononuclear phagocyte system, particularly in the liver and spleen, after repeated administration [213]. Additionally, interactions between MSNs and the immune system may trigger complement activation, macrophage activation, inflammatory cytokine release, hemocompatibility issues, and, in susceptible individuals, complement activation-related pseudoallergy. Consequently, comprehensive long-term studies on pharmacokinetics, biodistribution, biodegradation, immunotoxicity, and repeated-dose safety are required to establish the biosafety and clinical translation potential of MSN-based nanotherapeutics.

Notwithstanding these benefits, MSNs’ possible toxicity is still a major worry for biomedical applications. Evidence shows that nanoparticle design, dosage, exposure duration, and delivery method all affect toxicity. High doses or prolonged exposure can induce oxidative stress, ROS production, mitochondrial dysfunction, inflammatory responses, apoptosis, and alterations in cellular metabolism [214]. Additionally, organ-specific toxicity and immunological reactions may result from MSN accumulation in mononuclear phagocyte system organs, such as the liver, spleen, lungs, and kidneys. ROS-mediated signaling, inflammasome activation, cytokine release, and immunological modulation are key mechanisms underlying the biological effects of MSNs [215]. However, when MSNs are properly constructed, several studies have demonstrated minimal cytotoxicity, confirming their promise as safe nanocarriers for biomedical applications.

Recent advancements have significantly improved MSN safety and translational potential. Surface modification with biocompatible polymers, polysaccharides, lipids, peptides, and targeted ligands enhances colloidal stability, reduces nonspecific protein adsorption, lowers hemolytic activity, and optimizes biodistribution. Additionally, MSNs’ modifiable physicochemical characteristics provide precise control over drug-release patterns, biological interactions, and degradation kinetics, enabling safer and more efficient nanomedicine platforms [216]. Available data show that toxicity can be considerably reduced while maintaining therapeutic efficacy through deliberate adjustments to MSN design, porosity, surface chemistry, and delivery settings. Overall, these developments show significant progress in improving MSN biocompatibility and safety, supporting their continued growth as viable drug-delivery and biomedical platforms.

Although smart MSNs generally exhibit favorable biocompatibility, their biological safety is strongly influenced by physicochemical characteristics, including particle size, morphology, pore architecture, surface charge, silica condensation, surface functionalization, dosage, and exposure duration. Unmodified MSNs can induce oxidative stress, complement activation, macrophage-mediated clearance, inflammatory cytokine secretion, hemolysis, and immune activation, which restrict their long-term clinical application [217]. To address these adverse effects, research has focused on rational surface engineering strategies, including PEGylation, chitosan, hyaluronic acid, zwitterionic polymers, biomimetic cell membrane coatings, biodegradable organosilica frameworks, and charge-neutral surface modifications. These modifications reduce protein corona formation, nonspecific protein adsorption, MPS recognition, and inflammatory responses, while enhancing colloidal stability, circulation time, biodegradation, and immune compatibility. Despite these advances, significant translational challenges persist. The in vivo fate of MSNs depends on their physicochemical properties, which affect pharmacokinetics, biodistribution, cellular uptake, clearance pathways, and biodegradation [218]. Although biodegradable silica frameworks are metabolized to orthosilicic acid, differences in degradation kinetics may prolong accumulation in the liver, spleen, lungs, and other reticuloendothelial organs after repeated administration, potentially causing chronic inflammation or tissue-specific toxicity. Comprehensive preclinical studies, including pharmacokinetic profiling, quantitative organ biodistribution, long-term biodegradation, immunotoxicity, repeated-dose toxicity, and tissue-specific safety assessments in clinically relevant animal models, are therefore required to establish the long-term biosafety and clinical translation potential of MSN-based nanotherapeutics.

## 8. Challenges, Limitations, and Future Directions

Mesoporous silica nanoparticles have made great strides in drug delivery, bioimaging, cancer therapy, antimicrobial treatment, and regenerative medicine, but several obstacles still impede their clinical translation. Physicochemical characteristics of MSNs, including particle size, shape, pore architecture, surface charge, and surface functionalization, influence their biological performance. Limited target specificity, off-target accumulation, immunological recognition, endosomal entrapment, and uneven biodistribution remain major problems, even though these characteristics can be modified to improve therapeutic efficacy. Furthermore, biological barriers such as the blood–brain barrier, extracellular matrix, and mononuclear phagocyte system may significantly limit nanoparticle transport to target organs. Structural features, increased biodegradability, enhanced targeting, and regulated release profiles must be rationally designed [219].

Recent advances in nanotechnology have introduced novel approaches to overcome these constraints and improve the performance of MSN-based systems. Surface engineering with biocompatible polymers, peptides, antibodies, and biomimetic coatings has enhanced colloidal stability, circulation time, immunological evasion, and target selectivity. Notably, cell membrane-coated biomimetic MSN platforms show better homologous targeting and biological compatibility [220]. Stimulus-responsive and transformable MSN systems that respond to pH, enzymes, redox conditions, magnetic fields, or other external stimuli have enabled more accurate and controlled drug delivery. Additionally, new technologies such as multifunctional hybrid nanoplatforms and intelligent nanosystems have increased MSN capabilities by improving tissue penetration, cellular uptake, and therapeutic accuracy [221]. Advances in controlled polymerization techniques have also enabled highly defined surface designs, improving control of nanoparticle–biological interactions and therapeutic efficacy.

Multifunctional smart MSN platforms that incorporate gatekeepers, targeting ligands, polymers, imaging agents, and multiple therapeutic cargoes, have enhanced therapeutic precision, controlled drug release, and targeted delivery, their increasing structural complexity poses substantial barriers to clinical translation. Integrating diverse functional components complicates chemistry, manufacturing, and controls (CMC) by requiring precise control of particle size, pore architecture, surface chemistry, ligand density, drug loading, and release kinetics, as well as consistent batch-to-batch reproducibility [106]. Additionally, these multifunctional nanoplatforms demand comprehensive analytical characterization, process validation, sterilization, stability assessment, and Good Manufacturing Practice (GMP)-compliant production to ensure product quality, safety, and efficacy. Adding multiple responsive elements and therapeutic agents further increases manufacturing costs, limits scalability, and introduces variability in physicochemical properties and biological performance. The lack of harmonized regulatory frameworks for complex nanomedicines also complicates quality assessment, safety evaluation, and regulatory approval by agencies such as the U.S. Food and Drug Administration (FDA) and the European Medicines Agency (EMA). Consequently, simplifying nanoplatform design while maintaining therapeutic efficacy is essential to enhance manufacturability and expedite clinical translation [222].

Future advances in MSN-based nanomedicine will likely be driven by advancements in smart biomaterials, precision medicine, artificial intelligence (AI), and scalable manufacturing technologies. AI- and machine learning-assisted methodologies can enable the rational optimization of MSN physicochemical properties, surface functionalization, drug-loading efficiency, targeting specificity, and therapeutic efficacy, while simultaneously reducing experimental costs and development timelines. However, successful clinical translation requires more than enhanced nanoparticle design. It requires robust, economically viable, large-scale manufacturing processes that consistently produce highly reproducible, GMP-compliant MSN formulations. Standardized quality control measures, validated characterization techniques, efficient purification protocols, sterilization procedures, and comprehensive long-term stability assessments must support this. Additionally, thorough pharmacokinetic, biodistribution, biodegradation, immunotoxicity, and long-term safety evaluations are essential to meet regulatory standards and facilitate clinical approval. Creating internationally harmonized regulatory guidelines for silica-based nanomedicines, along with close collaboration among materials scientists, pharmaceutical engineers, clinicians, industry stakeholders, and regulatory authorities, is critical to bridging the gap between laboratory research and the successful clinical implementation of MSN-based therapeutics.

## 9. Conclusions

Mesoporous silica nanoparticles have emerged as versatile and promising nanoplatforms for advanced biomedical applications because of their unique physicochemical properties, including high surface area, tunable pore architecture, excellent drug-loading capacity, controllable biodegradation, and facile surface functionalization. These features enable the effective encapsulation and targeted administration of a variety of therapeutic agents, including proteins, peptides, nucleic acids, growth factors, antibacterial agents, and small-molecule medications. Recent developments in MSN engineering have enabled controlled, sustained, and stimuli-responsive drug delivery systems with enhanced therapeutic efficacy and reduced systemic toxicity. Additionally, by regulating drug release, enhancing cellular interactions, promoting angiogenesis and tissue regeneration, and integrating diagnostic and therapeutic functions into a single nanoplatform, MSN-based platforms have demonstrated considerable promise in cancer therapy, antimicrobial treatment, biofilm eradication, tissue engineering, regenerative medicine, and wound healing.

Despite these significant advances, several critical challenges must be addressed before MSN-based nanomedicines can achieve widespread clinical translation. Although numerous studies have shown promising in vitro performance, the ability to translate these findings into clinical applications remains limited because of the complexity of human physiology and the lack of predictive preclinical models. Comprehensive evaluation of long-term biocompatibility, biodegradation, biodistribution, immunogenicity, pharmacokinetics, large-scale manufacturing, reproducibility, and regulatory standardization remains essential. Future developments in surface engineering, biomimetic functionalization, intelligent stimuli-responsive systems, multifunctional theragnostic platforms, and rational nanoparticle design are expected to further improve the safety, targeting accuracy, and therapeutic efficacy of MSNs. Furthermore, standardized safety assessment protocols, clinically relevant preclinical models, and well-designed clinical trials should be prioritized to bridge the gap between laboratory discoveries and clinical implementation. Continued interdisciplinary collaboration among materials scientists, biomedical studies, clinicians, and regulatory agencies will be crucial for accelerating the translation of smart MSNs into precision medicine, regenerative therapies, and advanced wound management.

## Figures and Tables

**Table 1:** Overview of recent advances in MSN-based antibacterial delivery systems. The table summarizes nanoparticle composition, antimicrobial agents, bacterial targets, and proposed mechanisms responsible for enhanced antibacterial activity.

S. No	Nanoparticle Formulation	Active Agent	Major Target Bacteria	Antibacterial Assay	Main Finding	Proposed Mechanism	Ref.
1.	Metal-incorporated MSNs	Zn/Ag ions	*S. aureus*, *E. coli*	MIC, biofilm inhibition	Antibacterial and antibiofilm activity	ROS generation + membrane disruption	[68]
2.	Ofloxacin-loaded MSNs	Ofloxacin	*S. aureus*, *E. faecalis*, *P. mirabilis*, *P. aeruginosa*	ZOI, biofilm inhibition assay	Enhanced antibiotic efficacy	Sustained drug release	[69]
3.	Lysozyme and Vancomycin-MSNs	Lysozyme + Vancomycin	*S. aureus*	MIC, MBC	Efficient eradication of intracellular MRSA	Cell targeting + membrane disruption	[92]
4.	Ag- Rutin-decorated MSNs	Ag	*S. aureus*, *E. coli*	ZOI, MIC, MBC	Significant antibacterial activity	Ag^+^ release + ROS	[74]
5.	MCM-48@APTES	*Melissa officinalis* extract	*S. aureus*, *C. albicans*, *E. coli*, *K. pneumoniae*	MIC, ZOI	Synergistic antibacterial effect	Controlled phytochemical release	[90]
6.	Ti-M@A	Antimicrobial peptide	*S. aureus*, *E. coli*, *P. aeruginosa* and *MRSA*	Antibiofilm assay	Reduced biofilm formation	Sustained peptide release	[95]
7.	Ag-functionalized MSN coatings	Silver	Gram-positive bacteria	Colony count	Long-lasting antibacterial coating	Ag ion release	[84]
8.	MSN@Zn-COOH	Zinc	*S. aureus*, *E. coli*	MIC	Improved antibacterial & hemostatic performance	Zn^2+^ release	[85]
9.	CHX-loaded MSNs + ZnO QDs	Chlorhexidine	Oral bacteria	Biofilm inhibition	Strong antibacterial orthodontic resin	Sustained CHX release	[82]
10.	Fe_3_O_4_@SiO_2_-GS&EPMO-Cur	Curcumin/Gentamicin	*E. coli*, *S. aureus*	MIC, ZOI	Synergistic antibacterial effect	Dual-drug release	[81]
11.	Plasma-functionalized Ag-MSNs	Silver	*S. aureus*, *E. coli*	Colony assay	Improved antibacterial efficacy	Controlled Ag release	[71]
12.	SPIONs-loaded MSNs	Antibiotics	*E. coli*	Biofilm inhibition	Synergistic anti-biofilm activity	Sustained antibiotic delivery	[107]

**Table 3:** Representative MSN-based wound healing platforms, with their nanoparticle formulation, therapeutic cargo, wound model, biological performance, and underlying wound repair mechanisms highlighted.

S. No.	MSN Formulation	Therapeutic Cargo	Wound Model	Biological Activity	Major Findings	Proposed Mechanism	Ref.
1.	GelMA/HAMA/MSN@AE hydrogel	*Artemisia argyi* extract	Chronic wound	Anti-inflammatory, wound healing	Improved collagen deposition and wound closure	Controlled phytochemical release and inflammation suppression	[65]
2.	Hyaluronic acid-gentamicin hydrogel/MSNs	Gentamicin	Infected wound	Antibacterial wound dressing	Accelerated healing with real-time wound monitoring	Sustained antibiotic release and pH-responsive sensing	[179]
3.	Thiolated MSN@L-arginine alginate/guar film	L-Arginine	Skin wound	Blood-compatible wound healing	Enhanced tissue regeneration and hemocompatibility	Nitric oxide generation and improved cell proliferation	[180]
4.	Silver-coated MSNs	Ciprofloxacin + siRNA	Infected wound	Antibacterial wound healing	Rapid wound closure and bacterial eradication	Gene silencing and sustained antibiotic release	[181]
5.	Biomimetic liquid crystal-MSN composite hydrogel	Hydrogel matrix	Soft tissue injury	Tissue regeneration	Improved tissue repair and mechanical integrity	Biomimetic extracellular matrix support	[182]
6.	Curcumin-loaded MSN/PCL nanofibers	Curcumin	Skin wound	Antioxidant wound healing	Enhanced collagen synthesis and re-epithelialization	Sustained curcumin release and ROS scavenging	[192]
7.	Carboxymethyl chitosan/gelatin/MSNs film	*Myrtus communis* extract	Skin wound	Wound dressing	Accelerated wound contraction and antibacterial protection	Controlled phytochemical release	[194]
8.	Rutin/ZnO/MSN nano-hydrogel	Rutin + ZnO	Mouse wound model	Wound healing	Faster wound closure and collagen formation	Antioxidant and antibacterial synergism	[196]
9.	Doxycycline-loaded chitosan/MSN patch	Doxycycline	Skin wound	Anti-inflammatory, wound dressing	Reduced infection and improved healing	Sustained antibiotic delivery	[197]
10.	Ag-decorated MSN/chitosan nanofibers	Rutin+Ag	Diabetic wound	Antibacterial wound healing	Enhanced granulation tissue formation	Silver ion release and collagen deposition	[199]
11.	Carboxymethyl cellulose-HBPSi-MSN	Clindamycin	Wound dressing	Antibacterial tissue repair	Sustained drug release and infection control	Controlled antibiotic release	[200]
12.	Poloxamer 407-HPMC K15 M-MSN gel	*Rosmarinus officinalis* extract	Acute wound	Herbal wound healing	Accelerated epithelial regeneration	Anti-inflammatory and wound healing effects	[201]
13.	Nanocellulose-based MSNs	Antimicrobial peptides	Wound infection	Antimicrobial wound dressing	Controlled drug release and therapeutic wound dressings	Sustained AMP release and localized infection control	[206]

## Data Availability

No new data were created or analyzed in this study. Data sharing is not applicable to this article.

## Supplementary Materials

The following supporting information can be downloaded at https://www.mdpi.com/article/10.3390/pharmaceutics18081044/s1.
