# Supplementary material for: Smart Mesoporous Silica Nanoparticle-Based Drug Delivery Systems: Recent Advances in Biomedical Applications, Wound Healing and Therapeutic Perspectives
Source: Pharmaceutics. 2026 Aug 21;18(8):1044. doi: 10.3390/pharmaceutics18081044 (PMC13517001; doi:10.3390/pharmaceutics18081044)
Supplement: Supplementary file 1 [file pharmaceutics-18-01044-s001.zip › pharmaceutics-4413849-supplementary.pdf]

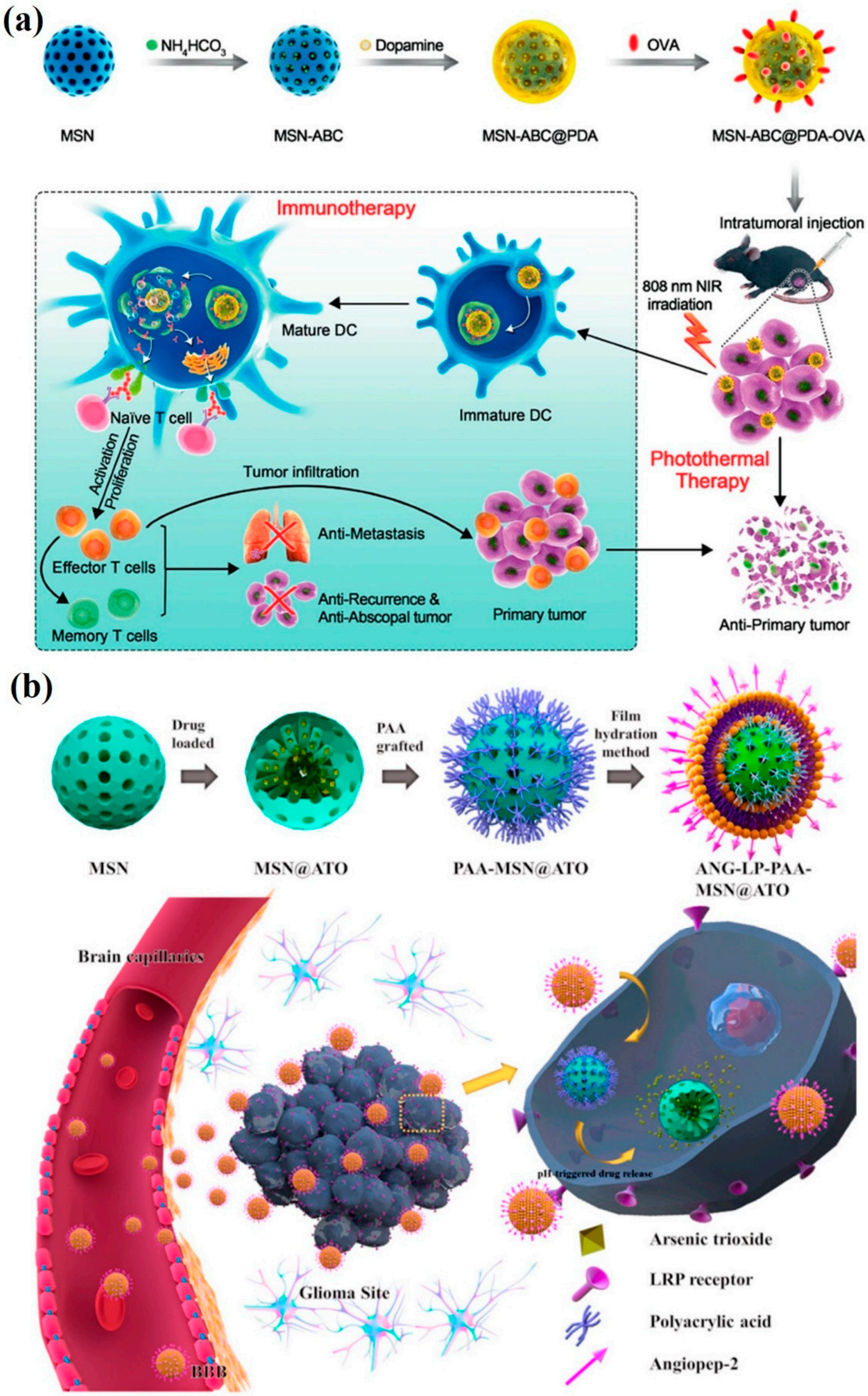

Figure S1: (**a**) MSNs-ABC@PDA-OVA nanovaccine was developed as a multifunctional mesoporous silica-based platform for melanoma treatment. The nanovaccine integrates photothermal therapy and antigen-mediated immunotherapy. Reproduced from [113] under the CCC License, Wiley Online Library. (**b**) Schematic illustration of angiopep-2-functionalized liposome–MSNs (ANG-LP-PAA-MSN) for targeted delivery of arsenic trioxide across the blood–brain barrier. The nanoplatform enables pH-responsive drug release, enhanced glioma accumulation, and improved therapeutic efficacy against intracranial tumors. Reproduced from [114] under the CCC License, Wiley Online Library.

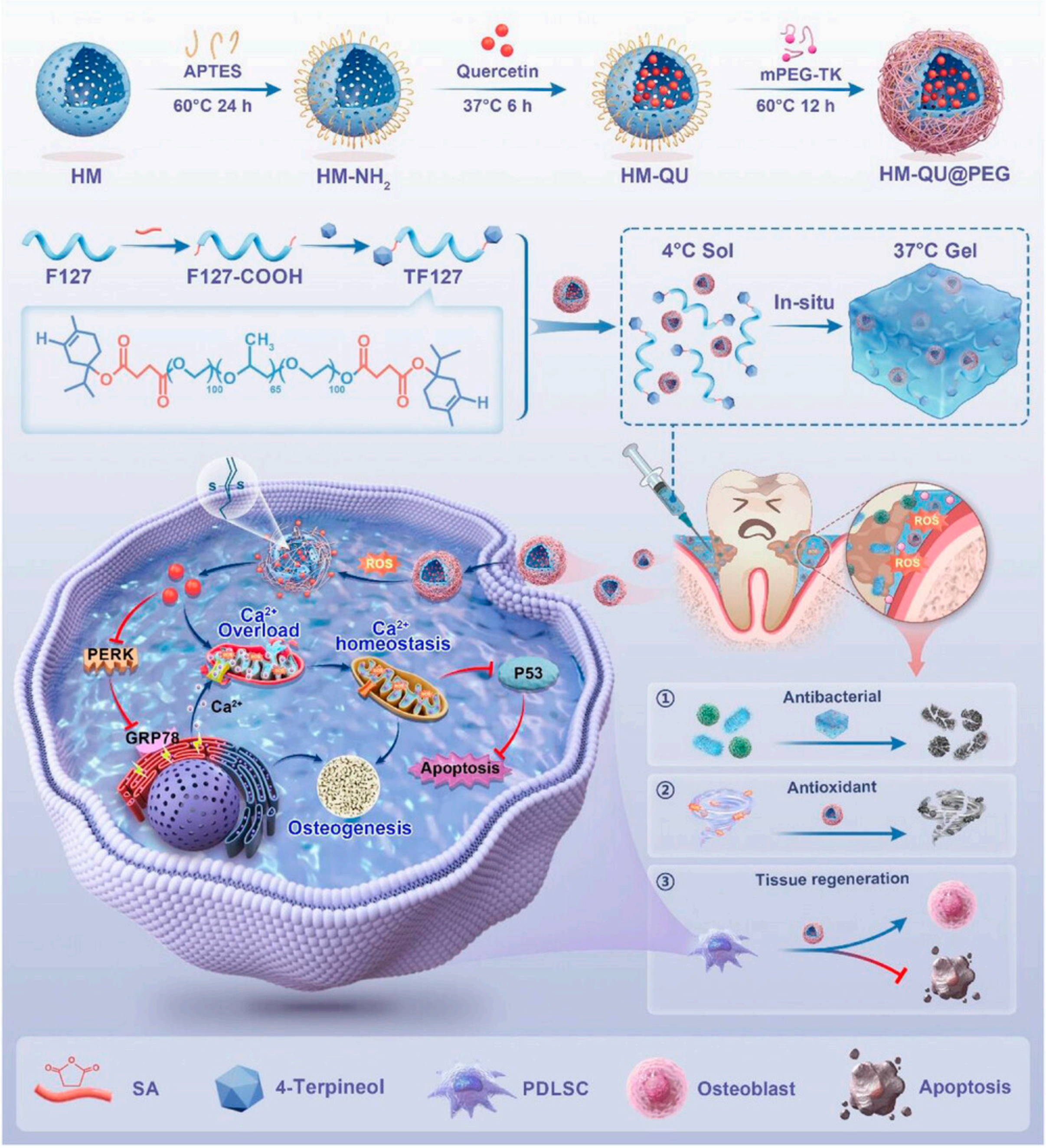

Figure S2: Schematic illustration of HQUP@TF127 embedded with quercetin-loaded hollow MSNs for periodontitis therapy. The platform eliminates periodontal pathogens, reduces oxidative stress and inflammation, promotes osteogenic differentiation of periodontal ligament stem cells, and enhances periodontal regeneration by inhibiting osteoclast activity and improving bone mineral density. Reproduced from [156], licensed under CC BY 4.0, Wiley Online Library.

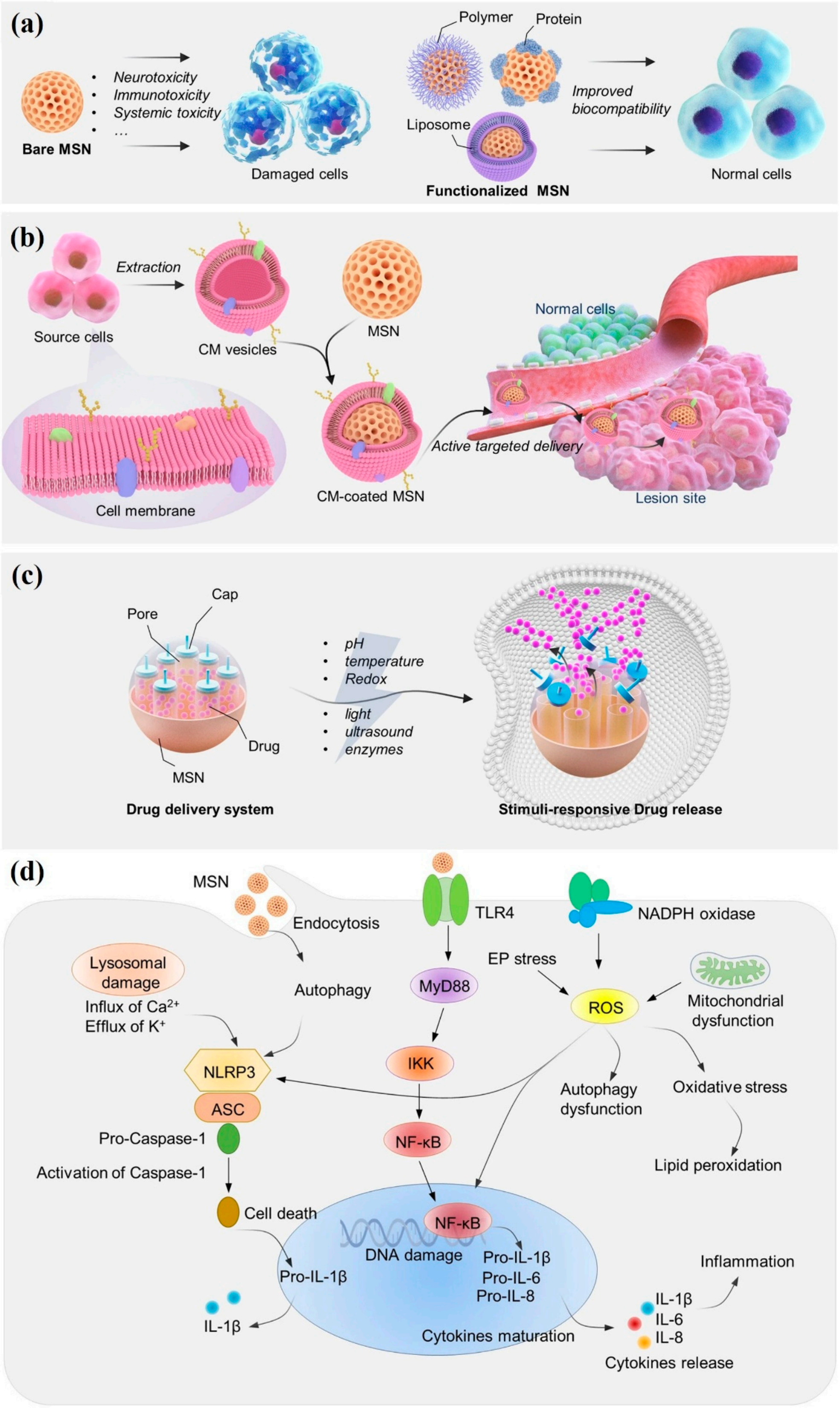

Figure S3. (**a**) Coating with polymers, proteins, or liposomes to enhance biocompatibility and reduce toxicity. (**b**) Cell membrane-based biomimetic modification to improve targeting capability and (**c**) incorporation of stimulus-responsive gatekeepers to enable controlled and site-specific drug release. (**d**) The primary mechanisms underlying silica nanoparticle-induced toxicity include oxidative stress, autophagy dysregulation, and pro-inflammatory responses. Reproduced from [176], licensed under CC BY 4.0, Springer Nature.

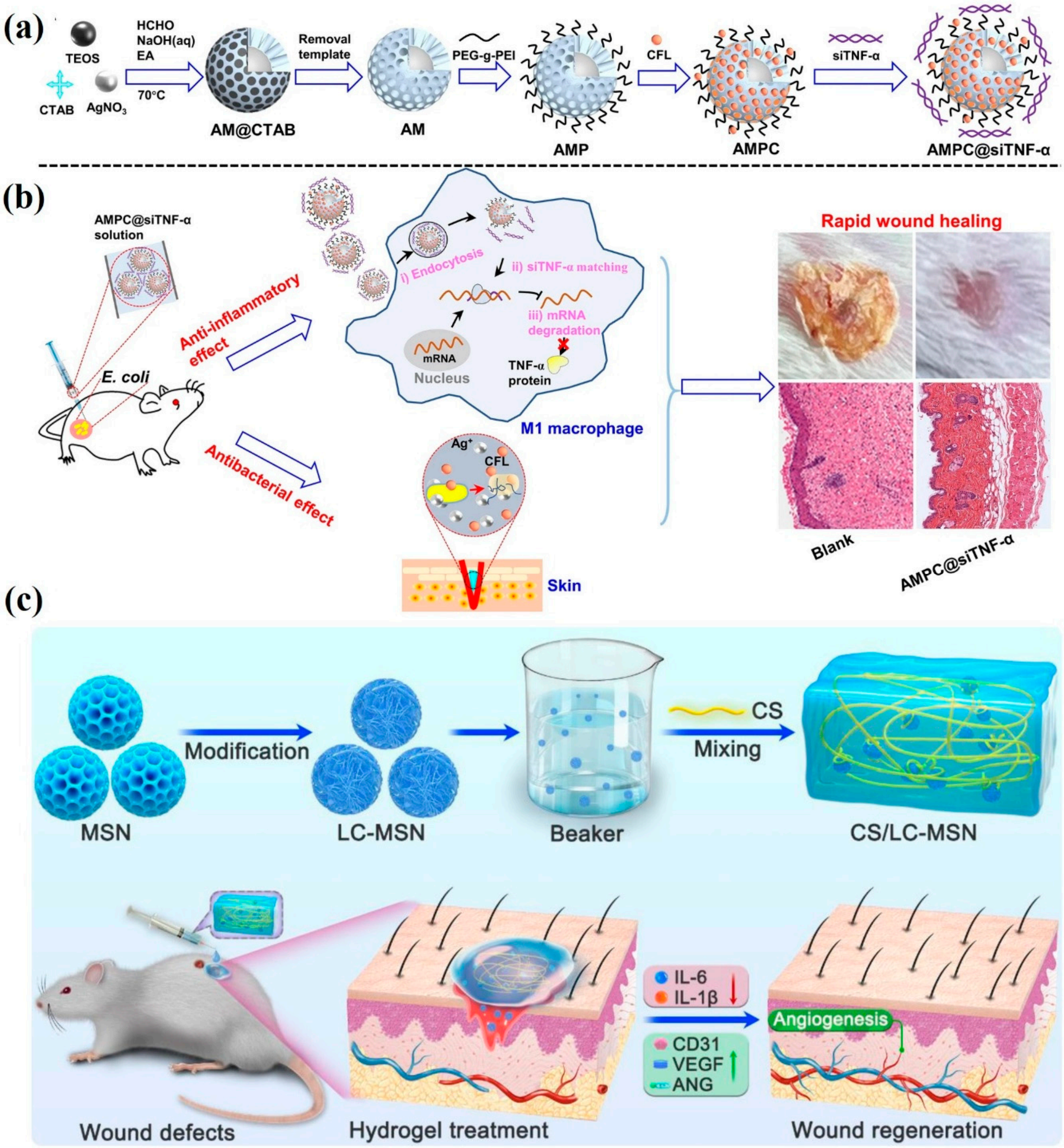

Figure S4: (**a**) Schematic illustration of the multifunctional AMPC@siTNF-α nanoplatform and (**b**) Mechanism of AMPC@siTNF-α-mediated infected wound healing, involving TNF-α gene silencing and synergistic promotion of tissue regeneration. Reproduced from [181], licensed under CC BY 4.0, Springer. (**c**) A schematic illustrates CS/LC–MSN, and an injectable CS/LC–MSN hydrogel enables minimally invasive administration to wound sites, offering a platform for wound healing and tissue regeneration. Reproduced from [182], licensed under CC BY 4.0, MDPI.
